# Transcriptome analysis of fenugreek under mixed saline-alkali stress

**DOI:** 10.3389/fpls.2026.1753943

**Published:** 2026-03-30

**Authors:** Jin Wang, Mingchuan Du, Haijuan Bao, Xiaoya Chen, Xiaojian Pu, Jiuli Wang, Jing Liu, Wei Wang

**Affiliations:** 1 College of Ecological Environment and Resources, Qinghai Minzu University, Xining, China; 2 Key Laboratory of Northwest Cultivated Land Conservation and Marginal Land Improvement Enterprises, Ministry of Agriculture and Rural Affairs, Delingha, China; 3 Qinghai Provincial Key Laboratory of High-Value Utilization of Characteristic Economic Plants, College of Ecological Environment and Resources, Qinghai Minzu University, Xining, China; 4 Qinghai Provincial Biotechnology and Analytical Test Key Laboratory, College of Ecological Environment and Resources, Qinghai Minzu University, Xining, China; 5 Academy of Animal Science and Veterinary Medicine, Qinghai University, Xining, China

**Keywords:** fenugreek, physiological responses, saline-alkali stress, seedling stage tolerance, transcriptome sequencing

## Abstract

**Background:**

Soil salinization and alkalization are major constraints to global crop production. Fenugreek (*Trigonella foenum-graecum* L.) is considered a potential salt-alkali-tolerant crop; however, the molecular mechanisms underlying its tolerance remain poorly understood.

**Methods:**

Four fenugreek provenances were evaluated under saline-alkali stress at the seedling stage. Growth and physiological traits were measured to assess stress tolerance. Based on these results, the most tolerant provenance was selected for transcriptome analysis. Saline-alkali treatments of 0 (control), 100 mmol/L (moderate stress), and 200 mmol/L (severe stress) were applied according to preliminary physiological experiments to induce measurable but non-lethal stress responses suitable for transcriptomic investigation.

**Results:**

The tolerance of the four provenances followed the order: Qinghai > Anhui > Yunnan > Gansu, with the Qinghai provenance showing the strongest tolerance. Transcriptome sequencing generated 47,757 unigenes, of which 27,300 were successfully annotated. A total of 2,041 differentially expressed genes (DEGs) were identified among treatments. Functional enrichment analysis indicated that these DEGs were mainly involved in metabolic processes, stress responses, and key pathways such as flavonoid biosynthesis, plant hormone signal transduction, and starch and sucrose metabolism. In addition, 24 transcription factors associated with abiotic stress were identified, primarily belonging to the AP2/ERF, bHLH, MYB, and NAC families. The reliability of the RNA-Seq results was confirmed by qRT-PCR analysis of nine selected DEGs using β-actin as the reference gene.

**Conclusions:**

These findings provide new insights into the molecular mechanisms underlying saline-alkali tolerance in fenugreek and offer valuable genetic resources for the breeding of stress-tolerant cultivars.

## 1 Introduction

Saline-alkali soil, as a global environmental issue, are increasingly exerting negative impacts on agricultural production and ecological sustainability (Lei et al., 2025). According to incomplete statistics, nearly 1 billion hectares of land worldwide are affected by saline-alkali conditions, accounting for approximately 7% of the global terrestrial area. This phenomenon impacts 50% of all irrigated land and 20% of arable land, thereby posing a serious threat to global crop yields and the development of a sustainable ecological environment (Liu and Wang, 2021).

Breeding salt-tolerant crops is a key strategy to mitigate the impact of soil salinization, and understanding the molecular responses of plants under saline-alkali stress is essential for genetic improvement. With the continuous advancement of scientific technologies, transcriptome sequencing (RNA-Seq) has been widely applied in the study of plant stress-resistance mechanisms, with its advantages of high throughput, reproducibility, and accuracy. Moreover, it does not require prior knowledge of a species’ genome and enables the analysis of transcriptomic information through *de novo* assembly (Livak and Schmittgen, 2001). By employing transcriptome sequencing, differentially expressed genes (DEGs) under saline-alkali stress can be identified, providing insights into stress-responsive genes and potential regulatory pathways. To date, transcriptome sequencing has been successfully applied to investigate gene expression under adverse conditions in several plants, including *Chenopodium quinoa* Willd (Bao et al., 2024), *Medicago sativa* L (Li et al., 2022), *Sorghum bicolor* (L.) Moench (Guo et al., 2023), and *Brassica napus* L (Campbell et al., 2015).

*Trigonella foenum-graecum* L., commonly known as fenugreek, is an annual herbaceous plant. In China, it is also called Xiangdouzi, Yunxiangcao, Xiangmuxu, Lubazi, and Kudou (Bakhtiar et al., 2024). Fenugreek contains various bioactive substances, such as flavonoids (Khenifi et al., 2023), saponins (Khalil et al., 2024; Kumar et al., 2025), and polysaccharides (Wang et al., 2024; Hu et al., 2025). The entire plant has medicinal uses and holds considerable medicinal (Haxhiraj et al., 2024), edible (Faisal et al., 2024), and forage (Zeng et al., 2024) value. As a traditional Chinese herbal remedy, fenugreek is widely grown in China. Moreover, it shows strong adaptability to different soils and climates, with notable cold and drought tolerance, indicating significant potential for development as a saline-alkali-tolerant species (Folina et al., 2024). To date, no research has utilized transcriptome sequencing to explore fenugreek’s response to saline-alkali stress. In this study, we first evaluated the saline-alkali tolerance of four fenugreek provenances (Qinghai, Anhui, Yunnan, and Gansu) through a comprehensive analysis of growth, physiological. The Qinghai provenance, identified as the most tolerant, was subsequently selected as the experimental material for transcriptome sequencing. Transcriptomic analysis was performed on its leaves exposed to varying levels of saline-alkali stress. Therefore, the objective of this study was to identify candidate genes associated with saline-alkali tolerance and to elucidate the underlying molecular mechanisms, thereby facilitating the breeding of cultivars with enhanced resistance.

## 2 Materials and methods

### 2.1 Plant materials

The seeds of four fenugreek provenances used in this study are listed in **Table 1**.

**Table 1:** Source of materials of fenugreek.

Code	Name	Source
A1	Qinghai fenugreek(QH)	The Huangyuan landrace of fenugreek, a locally adapted, traditional cultivar from Qinghai Province, China.
A2	Gansu fenugreek(GS)	The Pingliang landrace of fenugreek, a locally adapted, traditional cultivar from Gansu Province, China.
A3	Yunnan fenugreek(YN)	The Xinglin Qiancaotang-sourced fenugreek, a locally cultivated variety from Yunnan Province, China.
A4	Anhui fenugreek(AH)	The Bozhou Yaowangtang-sourced fenugreek, a locally cultivated variety from Anhui Province, China.

### 2.2 Experimental procedures

#### 2.2.1 Saline-alkali treatment

To ensure that the saline–alkali treatment reflected local field soil chemistry, surface soils (0–20 cm) were collected from 13 representative saline–alkali sites in Qinghai Province, China. Soil extracts were prepared following standard procedures, and the concentrations of major ions (CO_3_^2-^, HCO_3_^-^, SO_4_^2-^, Cl^-^, Na^+^, K^+^, Mg^2+^, and Ca^2+^). Based on the ionic composition profiles together with soil pH, hierarchical cluster analysis classified these sites into seven typical saline–alkali soil types. Across the dominant clusters, HCO_3_^-^ was consistently the predominant anion, followed by SO_4_^2-^ and Cl^-^, whereas CO_3_^2-^ occurred at relatively low proportions.

Based on the averaged molar ratios of the dominant anions observed in field soils, a representative bicarbonate-dominated mixed salt solution was prepared using Na_2_CO_3_, Na_2_SO_4_, NaCl, and NaHCO_3_ at a molar ratio of 1:3:3:9. Four stress levels were applied at total salt concentrations of 50, 100, 150, and 200 mmol L^-^¹ (Su et al., 2024). Control plants were irrigated with the same volume of distilled water as described above. Electrical conductivity (EC) and pH of each treatment solution were measured at 25°C using a conductivity meter and a pH meter, respectively; the corresponding EC and pH values are provided in **Table 2**.

**Table 2:** Concentration, EC and pH of the stress solutions.

Code	Concentration (mmol/L)	pH	EC
B1	0	7.47	0.00
B2	50	8.36	5.22
B3	100	8.32	9.75
B4	150	8.28	13.89
B5	200	8.24	17.79

#### 2.2.2 Measurement of seedling growth, photosynthetic, and physiological parameters

Seeds of fenugreek were sown in plastic pots (23.5 cm in diameter) containing a substrate mixture of vermiculite and perlite at a 4:1 (v/v) ratio. Before sowing, the substrate was thoroughly saturated with nutrient solution, and the pots were placed in a greenhouse. Seedlings were irrigated with pure water every two days. Upon reaching the three-leaf-one-heart stage, seedlings were randomly assigned to either a control group or various mixed saline-alkali stress treatment groups. The control group was irrigated with distilled water, while the treatment groups were irrigated with different concentrations of the saline-alkali stress solutions. Each treatment was replicated five times. After 20 days of stress exposure, fresh tissue samples were collected and stored at – 80 °C for subsequent analysis.

Growth measurements: Following the saline-alkali stress treatment, the plant height (shoot length) of fenugreek seedlings was measured. For each replicate, five plants were randomly selected, and their average height was recorded. Another set of five plants per replicate was then harvested. The aboveground parts were weighed to obtain the fresh weight. Subsequently, the samples were placed in an oven and heated at 105 °C for 20 minutes to inactivate enzymes, followed by drying at 80 °C until a constant weight was achieved to determine the dry biomass.

The following physiological parameters were measured using commercial assay kits (Nanjing Jisihuiyuan Biotechnology Co., Ltd., China) according to the manufacturer’s instructions: malondialdehyde (MDA), proline (Pro), soluble protein, and soluble sugar contents, as well as the activities of superoxide dismutase (SOD), peroxidase (POD), and catalase (CAT).

#### 2.2.3 Experimental design and sample collection for RNA-seq analysis

Based on the physiological evaluation of the aforementioned four fenugreek accessions, the one demonstrating the highest comprehensive salt-alkali tolerance was selected for this study. A pot experiment was conducted under controlled indoor conditions to simulate saline-alkali stress. Seeds were sown in plastic pots containing a substrate composed of vermiculite and perlite at a 4:1 (v/v) ratio. Before sowing, the substrate was saturated with Hoagland’s nutrient solution and subsequently irrigated with pure water every two days. Upon reaching the three-leaf-one-heart stage, seedlings were randomly assigned to either a control group or saline-alkali treatment groups. The control group was irrigated with pure water, while the treatment groups were subjected to moderate (100 mmol/L) and high (200 mmol/L) saline-alkali stress. Both control and treatment groups consisted of three biological replicates. After 20 days of treatment, a total of nine samples were collected. The third and fourth fully expanded leaves exhibiting uniform growth were selected from each plant, immediately frozen in liquid nitrogen, and stored at – 80 °C for subsequent RNA sequencing.

#### 2.2.4 Total RNA extraction, cDNA synthesis, and transcriptome sequencing

Total RNA was extracted from fenugreek leaves using the TRIzol reagent. RNA purity and concentration were determined with a NanoDrop 2000 spectrophotometer, and RNA integrity was assessed using an Agilent 2100 Bioanalyzer/LabChip GX system. After quality control, mRNA was enriched using Oligo(dT) magnetic beads and subsequently fragmented into short sequences. The fragmented mRNA was used as a template for first-strand cDNA synthesis, followed by purification. The purified cDNA was then end-repaired, adaptor-ligated, and PCR-amplified to construct cDNA libraries. Sequencing was performed on the Illumina NovaSeq 6000 platform (Biomarker, Beijing, China) with paired-end 150 bp (PE150) reads. After filtering and quality control, high-quality clean reads were obtained. The clean reads were assembled *de novo* using Trinity software, and redundant sequences were removed to generate a set of unigenes.

#### 2.2.5 Functional annotation of unigenes and expression quantification

Unigene sequences were aligned against the NR, Swiss-Prot, COG, KOG, eggNOG4.5, and KEGG databases using DIAMOND software. KEGG Orthology (KO) assignments were obtained with KOBAS. Gene Ontology (GO) annotations for novel genes were analyzed using InterProScan by leveraging the integrated InterPro database. Following the prediction of amino acid sequences, unigenes were aligned to the Pfam database using HMMER software to obtain functional annotations.

Sequencing reads were mapped to the unigene library using Bowtie (Langmead et al., 2009). Based on the alignment results, expression levels were estimated with RSEM (Li and Dewey, 2011). The abundance of each unigene was quantified using the FPKM (Fragments Per Kilobase of transcript per Million mapped reads) value (Trapnell et al., 2010).

#### 2.2.6 Screening and functional annotation of differentially expressed genes

Differential expression analysis was carried out using DESeq2, with genes exhibiting a fold change ≥ 2 and a false discovery rate (FDR)< 0.01 identified as DEGs (Qi, 2018). For exploratory data analysis and visualization, count data were variance-stabilized (vst, DESeq2) and used for PCA and sample-to-sample distance clustering.

GO enrichment analysis of the DEGs was performed using the topGO package. KEGG pathway enrichment analysis was conducted using KOBAS 2.0.

The raw sequencing data generated in this study have been deposited in the Genome Sequence Archive (Chen et al., 2021) at the National Genomics Data Center (CNCB-NGDC Members and Partners, 2025), China National Center for Bioinformation/Beijing Institute of Genomics, Chinese Academy of Sciences, under accession number CRA030543.

#### 2.2.7 qRT-PCR confirmation of candidate genes

To confirm the reliability of the transcriptome results, nine DEGs showing significant expression changes were randomly chosen for validation by qRT-PCR. The IDs of these DEGs along with their primer sequences are provided in **Table 3**. *β-actin* served as the internal control gene (Ma et al., 2022). Three technical replicates were set up for each gene in every sample, and relative expression levels were determined using the 2^−^*^ΔΔCt^* method (Wang et al., 2009).

**Table 3:** Primers used for the qRT-PCR validation.

Gene ID	Forward primer	Reverse primer
*CCCH20*	GACACCGTATCGGGACTGC	TTGTAACGACGGAACCTATTGT
*NF-YB3L*	GAGCAACCACCGTCAAACC	CTCCTCCCGATTCGTTGTC
*RAP2-9*	TCAGCCGTCACATAACCTCC	GGGTTCTGAGTGGGATGTTAGT
*COL5*	AGTTATTATTCTGGAAGTTGTTTGAGT	AGACGACGCCGAAGCAG
*MYB-HBL1*	GACCATCTGTAATAATCACACATAGC	GATGGTCGTGATTGCGTTG
*bZIP53*	CCATCTTCACATACGCTTCCTC	AATCAGCTCGACGATCCAGAA
*ERF6*	AAGAGGAAGAGGTGGTAGAGGTAA	CGACGAAGGAACGCTGAA
*TIFY 3B*	TGCTGCCGCTGCCAAG	GCCTGCGGTGAAGCGAT
*Obf1-like*	CGGCGGTCTAGGATGAGG	CCAACCTATTACTCAACTCACCC
*β-actin*	TCGCTGCTGAGGTTTTGGAA	CCAATTTCGCCTTTGCCCTT

#### 2.2.8 Statistical analysis

Microsoft Excel 2025 was used for data organization and transformation (arcsine transformation for percentage data and logarithmic transformation where appropriate). Figures were prepared using Origin 2024. Statistical analyses were conducted using SPSS 21.0 Prior to analysis, normality was tested and homogeneity of variance was assessed using Levene’s test. A two-way analysis of variance (ANOVA) was performed to evaluate the effects of germplasm, treatment concentration, and their interaction (germplasm × concentration) on growth and physiological parameters. When significant effects were detected (*P* < 0.05), means were compared using Duncan’s multiple range test. In figures, different lowercase letters indicate statistically significant differences among treatments at *P* < 0.05. Comprehensive evaluation across multiple traits was performed using the membership function method (Zhu et al., 2024).

Formula for the comprehensive evaluation of saline–alkali tolerance (Equations 1, 2):

(1) $$\mu_{1}(X_{j})=\frac{X_{j}-X_{\min}}{X_{\max}-X_{\min}},\;j=1,2,\text{……},n$$

(2) $$\mu_{2}(X_{j})=1-\mu_{1}(X_{j}),\;j=1,2,\text{……},n$$

Where μ(X_j_) represents the membership function value of the j-th indicator; X_j_ denotes the measured value of the j-th indicator; X_min_ is the minimum value of the j-th indicator; and X_max_ is the maximum value of the j-th indicator.

Formula for calculating the weights of the evaluation indicators (Equation 3):

(3) $$W_{j}=\frac{P_{j}}{\sum_{j-1}^{n}P_{j}},\;j=1,2,\text{……},n$$

Where W_j_ represents the importance (i.e., weight) of the j-th measured indicator among all indicators, and P_j_ represents the contribution rate of the j-th indicator for each material (accession).

Formula for calculating the comprehensive evaluation value of saline–alkali tolerance (Equation 4):

(4) $$D=\sum_{j}^{n}\mu (X_{j})*W_{j},\;j=1,2,\text{……},n$$

Where D denotes the comprehensive evaluation value of saline-alkali tolerance for all indicators.

## 3 Results

### 3.1 Analysis of variance

#### 3.1.1 Growth parameters

As shown in **Table 4**, analysis of variance revealed highly significant differences (*P* < 0.01) in the shoot dry weight and plant height of fenugreek seedlings among germplasms and under different saline–alkali stress treatments. These traits were identified as effective indicators reflecting the response of fenugreek seedlings to saline–alkali stress and were therefore subjected to further multiple comparison analysis.

**Table 4:** Analysis of variance of growth index of tested fenugreek materials.

Source of variation	F-value	
	Dry weight	Plant height
Between provenances	18.32**	76.38**
Stress	193.12**	132.31**
Provenance×Stress	1.08	1.22

* and ** refer to the different significance at 0.05 and 0.01 levels, respectively, the same below.

#### 3.1.2 Physiological parameters

As shown in **Table 5**, the contents of malondialdehyde (MDA), proline (Pro), soluble sugars, and soluble proteins, as well as the activities of superoxide dismutase (SOD), peroxidase (POD), and catalase (CAT), exhibited significant (*P* < 0.05) or highly significant (*P* < 0.01) differences among germplasms, saline–alkali stress levels, and their interactions. These physiological parameters were identified as key indicators of the saline–alkali stress response in fenugreek seedlings and were further examined by multiple comparison analysis.

**Table 5:** Analysis of variance of physiological indexes of fenugreek materials under saline-alkali stress.

Source of variation	F-value						
	MDA	Pro	SOD	POD	CAT	Soluble sugar	Soluble protein
Provenance	.77**	175.53*	25.30*	74.24*	95.21*	28.90*	903.52*
Stress	61.76*	110.64*	12.14*	53.20*	282.84*	157.10*	163.63*
Provenance×Stress	4.89*	4.48*	5.18*	21.80*	19.04*	12.23*	18.00*

### 3.2 Effects of mixed saline-alkali stress on the growth of fenugreek seedlings

**Figure 1:**
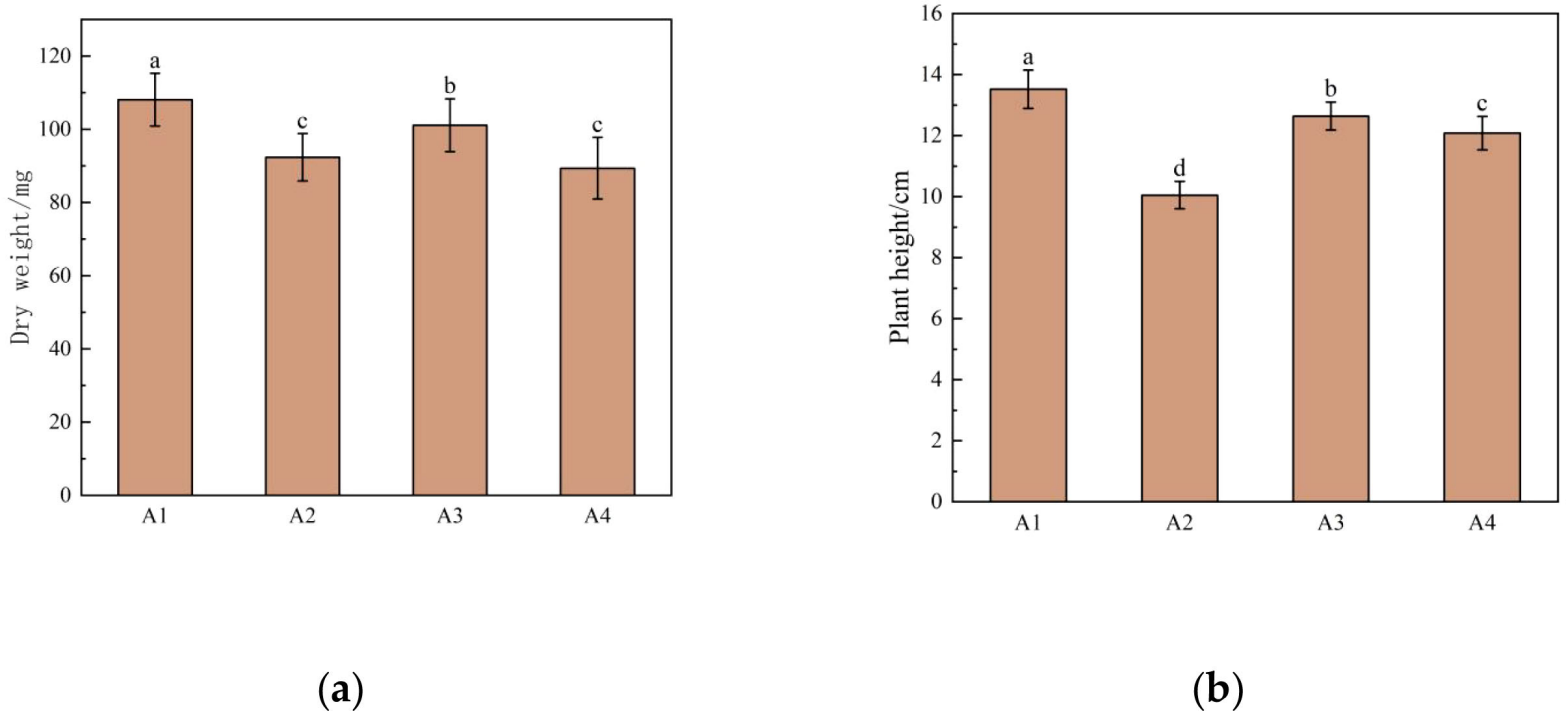
Effects of saline-alkali stress on growth indexes of fenugreek seedlings among provenances. **(a)** Dry weight; **(b)** Plant height. A1, A2, A3 and A4 represent Qinghai fenugreek, Gansu fenugreek, Yunnan fenugreek and Anhui fenugreek, respectively.

As shown in **Figure 1**, the shoot dry weight and plant height of fenugreek seedlings varied among germplasms under mixed saline-alkali stress. The Qinghai fenugreek exhibited the highest shoot dry weight (108.09 mg) and plant height (13.52 cm), which were significantly higher than those of the other germplasms (*P* < 0.05). The Yunnan fenugreek also showed relatively high shoot dry weight (101.11 mg) and plant height (12.64 cm), significantly exceeding those of the Gansu (92.37 mg, 10.05 cm) and Anhui (89.39 mg, 12.08 cm) germplasms (*P* < 0.05).

**Figure 2:**
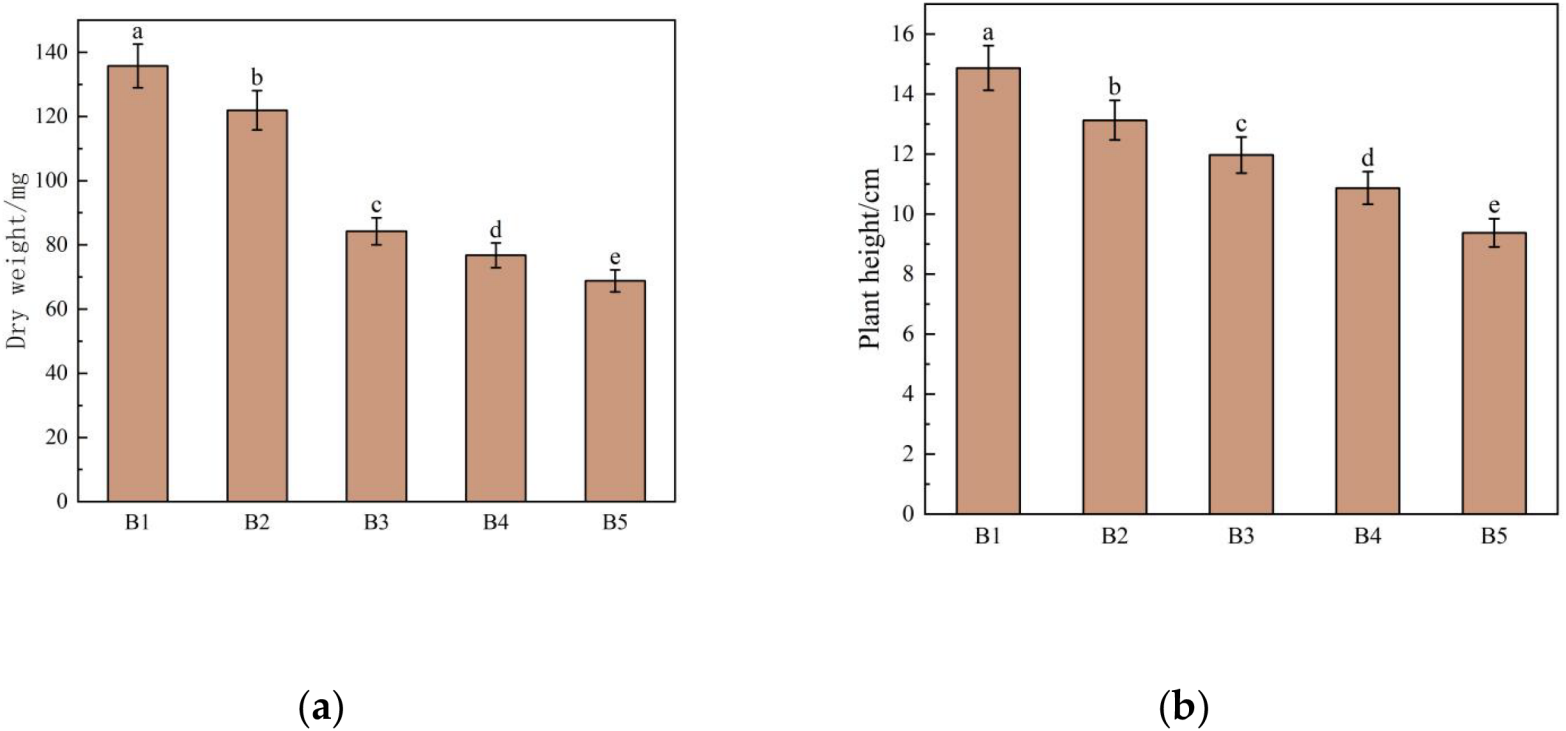
Effects of different concentrations of saline-alkali stress on growth indexes of fenugreek seedlings. **(a)** Dry weight; **(b)** Plant height. B1, B2, B3, B4 and B5 represent the saline-alkali concentration of 0 mmol/L, 50 mmol/L, 100 mmol/L, 150 mmol/L and 200 mmol/L, respectively.

Plant height and biomass accumulation serve as the most direct indicators of plant growth under saline-alkali stress. Under such conditions, plants may experience osmotic stress, nutrient deficiency, oxidative damage, and metabolic disorders, all of which hinder biomass accumulation (Zhang et al., 2025). As shown in **Figure 2**, both shoot dry weight and plant height of fenugreek seedlings decreased with increasing saline-alkali stress intensity. The shoot dry weights in treatments B2, B3, and B4 were 121.96 mg, 84.24 mg, and 76.77 mg, representing reductions of 10.17%, 37.95%, and 43.46%, respectively, compared with the control (B1, 135.77 mg). Similarly, plant heights in B2, B3, and B4 were 13.13 cm, 11.97 cm, and 10.87 cm, which were 11.70%, 19.50%, and 26.90% lower than those of the control (B1, 14.87 cm).

### 3.3 Effects of mixed saline-alkali stress on physiological indices of fenugreek seedlings

**Figure 3:**
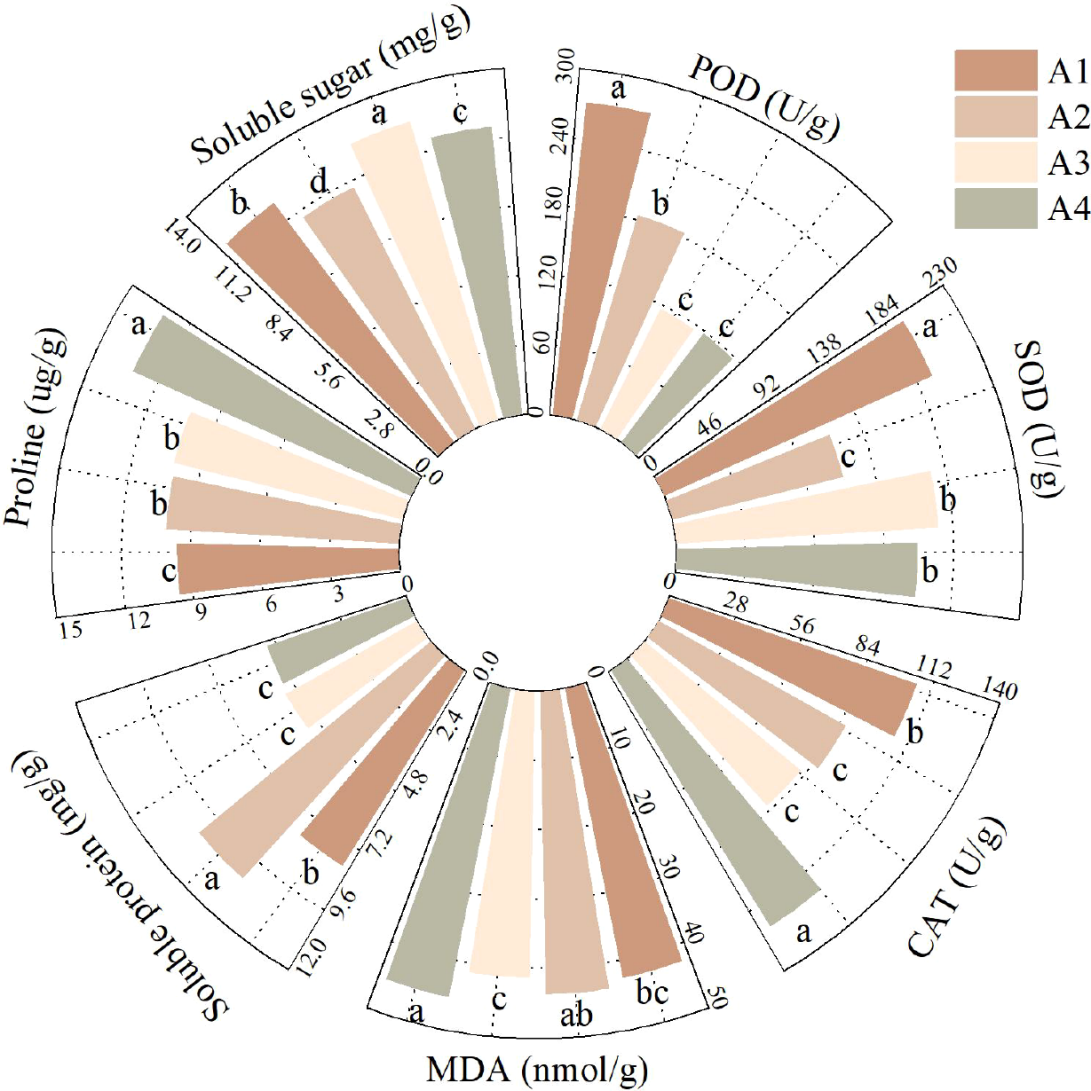
Effects of mixed saline-alkali stress on physiological indexes of fenugreek seedlings among provenances.

As shown in **Figure 3**, significant differences were observed in the physiological indices of fenugreek seedlings from different provenances under mixed saline-alkali stress. The Qinghai fenugreek displayed the highest peroxidase (POD) activity (272.00 U/g), which was significantly higher than that of all other provenances (*P* < 0.05). The Gansu fenugreek had relatively high POD activity (184.31 U/g), significantly surpassing that of the Yunnan (115.97 U/g) and Anhui (118.00 U/g) provenances (*P* < 0.05).

Similarly, the Qinghai fenugreek exhibited the highest superoxide dismutase (SOD) activity (194.82 U/g), significantly exceeding that of all other provenances (*P* < 0.05). The Yunnan fenugreek showed comparatively high SOD activity (174.08 U/g), which was not significantly different from that of the Anhui provenance (159.81 U/g) but was significantly higher than that of the Gansu provenance (116.75 U/g) (*P* < 0.05).

The catalase (CAT) activity peaked in the Anhui fenugreek (121.52 U/g), which was significantly higher than that in all other provenances (*P* < 0.05). The Qinghai fenugreek also demonstrated relatively high CAT activity (105.90 U/g), significantly exceeding that of the Gansu (86.64 U/g) and Yunnan (81.78 U/g) provenances (*P* < 0.05).

**Figure 4:**
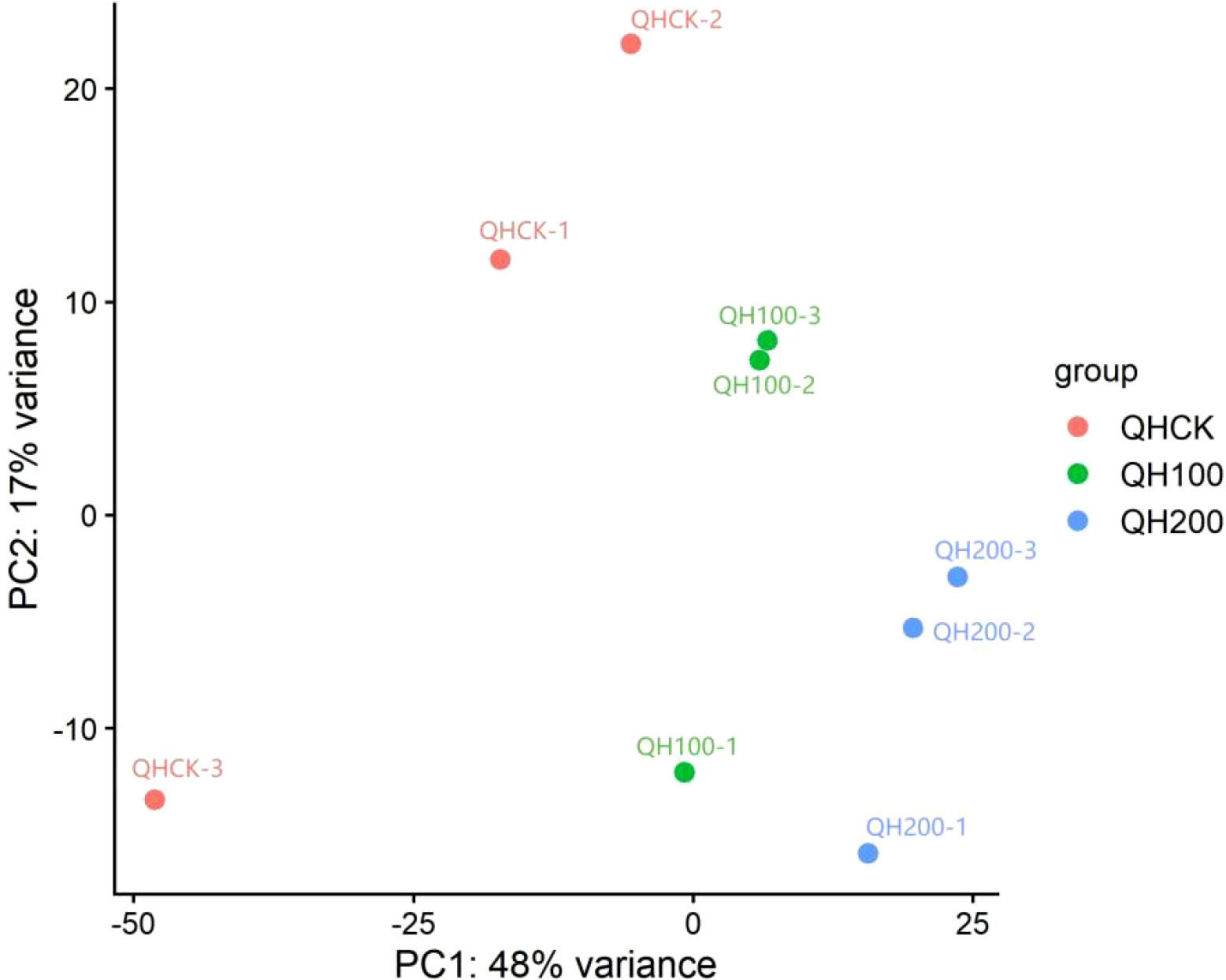
Principal component analysis (PCA) of fenugreek leaf transcriptomes under saline-alkali stress.

Malondialdehyde (MDA), a byproduct of membrane lipid peroxidation, serves as an indicator of the intensity of saline-alkali stress in plants (Li and Li, 2009). As illustrated in **Figure 4**, the Yunnan fenugreek had the lowest MDA content (41.26 nmol/g), which was not significantly different from that of the Qinghai provenance (42.45 nmol/g) but was significantly lower than that of the Gansu (43.56 nmol/g) and Anhui (45.24 nmol/g) provenances (*P* < 0.05). The Qinghai provenance also exhibited a relatively low MDA content, which was significantly lower than that of the Anhui provenance (*P* < 0.05).

Osmotic adjustment is crucial for water uptake in plants under saline-alkali stress (Zhao et al., 2025). The Gansu fenugreek showed the highest soluble protein content (10.39 mg/g), significantly exceeding that of all other provenances (*P* < 0.05). The Qinghai fenugreek had a relatively high soluble protein content (7.95 mg/g), which was significantly greater than that of the Yunnan (5.19 mg/g) and Anhui (5.09 mg/g) provenances (*P* < 0.05).

The Anhui fenugreek exhibited the highest proline content (13.16 mg/g), significantly surpassing that of the other provenances (*P* < 0.05). No significant difference was observed in proline content between the Gansu (10.09 mg/g) and Yunnan (10.19 mg/g) provenances, yet both were significantly higher than that of the Qinghai provenance (9.58 mg/g) (*P* < 0.05).

The soluble sugar content was highest in the Yunnan fenugreek (12.55 mg/g), significantly greater than that of other provenances (*P* < 0.05). The Qinghai fenugreek also had a relatively high soluble sugar content (12.09 mg/g), which was significantly higher than that of the Gansu (10.91 mg/g) and Anhui (11.69 mg/g) provenances (*P* < 0.05).

**Figure 5:**
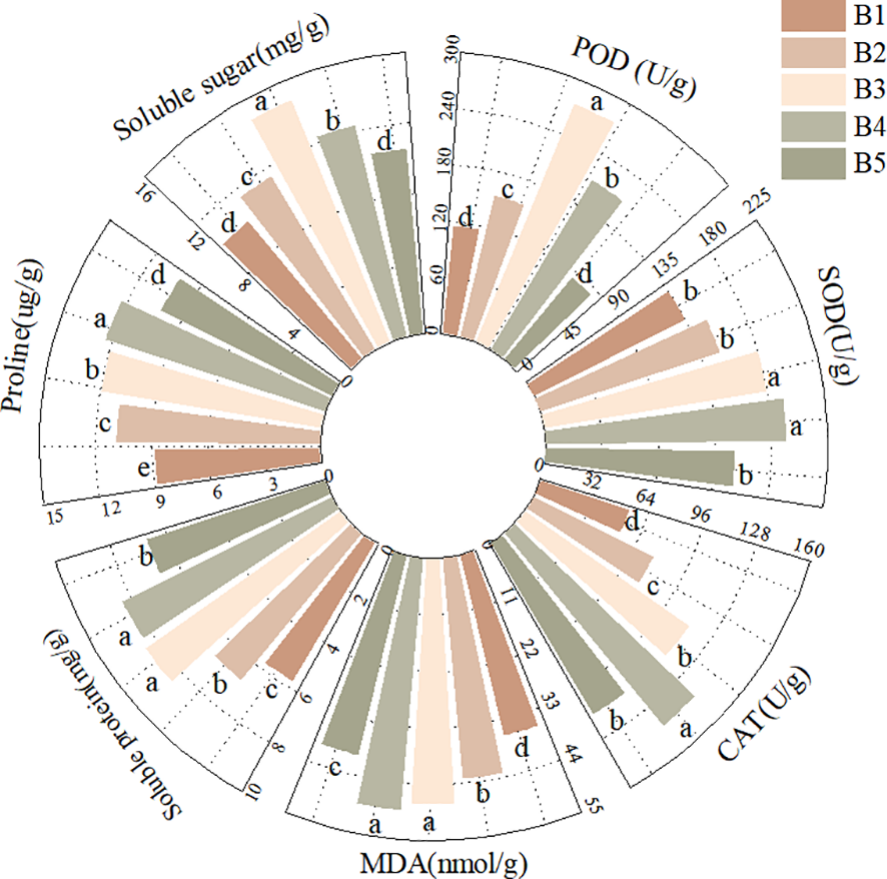
Effects of different concentrations of saline-alkali stress on physiological indexes of fenugreek seedlings.

As shown in **Figure 5**, the activities of antioxidant enzymes, contents of osmotic adjustment substances, and MDA content in fenugreek exhibited a trend of an initial increase followed by a decrease with increasing mixed saline-alkali stress concentration, peaking under either B3 or B4 treatments.

The highest peroxidase (POD) activity (274.44 U/g), soluble sugar content (14.77 mg/g), and soluble protein content (8.46 mg/g) were all observed under the B3 treatment. The POD activity and soluble sugar content at B3 were significantly higher than those in all other treatments (*P* < 0.05). The soluble protein content at B3 showed no significant difference from that at B4 (8.32 mg/g), but both were significantly higher than in the other treatments (*P* < 0.05).

In contrast, the highest proline content (12.31 mg/g), superoxide dismutase (SOD) activity (192.05 U/g), and catalase (CAT) activity (141.36 U/g) were recorded under the B4 treatment. The CAT activity and proline content at B4 significantly surpassed those in other treatments (*P* < 0.05). The SOD activity at B4 showed no significant difference from that at B3 (179.76 U/g), yet both were significantly higher than in the other treatments (*P* < 0.05).

As the stress concentration increased, the MDA content first increased and then decreased, reaching its lowest value under B5 treatment (39.95 nmol/g), which was significantly lower than that in all other treatments (*P* < 0.05). This reduction may be attributed to the elevated activities of antioxidant enzymes and increased contents of osmotic adjustment substances, which likely alleviated the damage induced by saline-alkali stress.

As presented in **Table 6**, the interaction between seed source and saline-alkali stress significantly influenced the physiological and biochemical parameters of fenugreek seedlings.

**Table 6:** Effects of provenance × stress on physiological indexes of fenugreek seedlings.

Level	POD (U/g)	SOD (U/g)	CAT (U/g)	MDA (nmol/g)	Soluble protein (mg/g)	Pro (ug/g)	Soluble sugar (mg/g)
A1B1	173.33 ± 6.67d	200.03 ± 33.52bcd	60.42 ± 3.88h	31.82 ± 0.38j	5.78 ± 0.02i	7.46 ± 0.11j	8.52 ± 0.02k
A1B2	246.67 ± 17.64c	188.54 ± 8.14bcde	79.10 ± 2.26g	46.96 ± 0.62c	7.53 ± 0.12fg	9.61 ± 0.14h	11.29 ± 0.07h
A1B3	340.00 ± 0.00b	184.37 ± 19.48bcdef	153.68 ± 2.26b	50.31 ± 0.31ab	10.22 ± 0.02c	10.38 ± 0.07f	16.78 ± 0.05a
A1B4	440.00 ± 46.19a	184.76 ± 20.86bcdef	135.60 ± 3.91c	44.12 ± 0.17d	8.58 ± 0.14e	11.95 ± 0.47cd	12.66 ± 0.07d
A1B5	160.00 ± 11.55de	216.42 ± 23.12abc	100.69 ± 3.88f	39.04 ± 0.09fg	7.64 ± 0.10f	8.50 ± 0.06i	11.18 ± 0.02h
A2B1	73.33 ± 13.33h	69.25 ± 1.68k	35.80 ± 2.24i	35.52 ± 1.68i	8.48 ± 0.27e	7.43 ± 0.25j	8.59 ± 0.03k
A2B2	133.33 ± 6.67defgh	98.49 ± 4.73ik	58.82 ± 4.85h	39.99 ± 0.97ef	9.61 ± 0.05d	10.32 ± 0.21fg	10.40 ± 0.07i
A2B3	486.67 ± 59.25a	134.53 ± 5.92ghij	103.78 ± 7.99ef	50.40 ± 0.45ab	11.98 ± 0.16b	11.68 ± 0.12cde	13.63 ± 0.05c
A2B4	134.68 ± 6.73defgh	161.37 ± 3.58defghi	121.39 ± 2.02d	51.77 ± 0.30a	12.34 ± 0.22a	11.27 ± 0.08e	12.71 ± 0.03d
A2B5	93.55 ± 6.25fgh	120.10 ± 3.53ij	113.42 ± 4.49de	40.59 ± 0.09ef	9.54 ± 0.12d	9.73 ± 0.12gh	9.23 ± 0.03j
A3B1	82.30 ± 0.95gh	111.99 ± 17.93ik	46.48 ± 3.82i	37.41 ± 0.15gh	3.51 ± 0.06m	7.88 ± 0.25j	11.65 ± 0.10gh
A3B2	118.30 ± 0.39defgh	144.78 ± 7.72efghij	68.07 ± 3.93gh	41.11 ± 0.31e	4.83 ± 0.03k	10.02 ± 0.12fgh	12.36 ± 0.09de
A3B3	131.55 ± 5.16defgh	221.21 ± 8.12ab	80.40 ± 3.87g	41.02 ± 0.45e	5.17 ± 0.03jk	11.35 ± 0.28de	14.45 ± 0.73b
A3B4	148.03 ± 7.06def	252.43 ± 11.32a	113.86 ± 3.31de	50.14 ± 0.17ab	7.30 ± 0.00g	12.15 ± 0.27c	12.30 ± 0.07def
A3B5	99.68 ± 0.63efgh	139.96 ± 7.57fghij	100.12 ± 2.42f	36.64 ± 0.30hi	5.16 ± 0.05jk	9.56 ± 0.12h	11.97 ± 0.06efg
A4B1	90.30 ± 5.96fgh	137.75 ± 7.80fghij	61.02 ± 3.91h	35.69 ± 0.82hi	4.35 ± 0.04l	12.04 ± 0.07c	11.22 ± 0.03h
A4B2	147.11 ± 17.69def	184.56 ± 24.18bcdef	97.04 ± 2.13f	46.27 ± 0.38c	5.25 ± 0.08j	12.92 ± 0.12b	11.69 ± 0.13fgh
A4B3	138.35 ± 0.70defg	178.92 ± 11.00bcdefg	115.91 ± 2.81de	49.71 ± 0.37b	6.45 ± 0.11h	14.18 ± 0.24a	14.23 ± 0.41b
A4B4	117.00 ± 0.57defgh	169.65 ± 8.10cdefgh	194.57 ± 8.35a	51.00 ± 0.42ab	5.07 ± 0.02jk	13.88 ± 0.18a	11.73 ± 0.03fgh
A4B5	97.22 ± 0.60fgh	128.19 ± 9.19hij	139.05 ± 2.20c	43.52 ± 0.56d	4.33 ± 0.02l	12.79 ± 0.32b	9.61 ± 0.03j

Regarding antioxidant enzyme activities, peroxidase (POD) ranged from 73.33 to 486.67 U/g, peaking in treatment A2B3 (486.67 U/g). This value was comparable to that in A1B4 (440.00 U/g) but was significantly higher than in all other treatments (*P* < 0.05). Superoxide dismutase (SOD) activity varied between 69.25 and 225.43 U/g, with the maximum observed in A3B4 (225.43 U/g), which did not differ significantly from A1B5 (216.42 U/g) or A3B3 (221.21 U/g) but was significantly higher than the remaining treatments (*P* < 0.05). Catalase (CAT) activity spanned from 35.80 to 194.57 U/g, reaching its highest level in A4B4 (194.57 U/g), a value that significantly exceeded all other treatments (*P* < 0.05).

For stress indicators and osmoregulatory substances, malondialdehyde (MDA) content ranged from 31.82 to 51.77 nmol/g. The lowest content was recorded in A3B5 (36.64 nmol/g) and was significantly lower than that in all other treatments (*P* < 0.05). Soluble protein content (3.51 - 12.34 mg/g) was highest in A2B4 (12.34 mg/g), significantly surpassing other treatments. Soluble sugar content (8.52 - 16.78 mg/g) peaked in A1B3 (16.78 mg/g), which was also significantly higher than in other treatments (*P* < 0.05). Finally, proline content ranged from 7.43 to 14.18 μg/g. The highest level was found in A4B3 (14.18 μg/g), which was not significantly different from A4B4 (13.88 μg/g) but was significantly greater than all other treatments (*P* < 0.05).

### 3.4 Comprehensive evaluation of the effects of saline-alkali stress on the growth of fenugreek seedlings from different provenances

Evaluating the saline-alkali tolerance of crops using a single indicator often yields inconsistent and incomparable results, making it challenging to accurately gauge plant tolerance. Therefore, a comprehensive evaluation based on multiple indicators is essential. In this study, a suite of fifteen growth, physiological from four fenugreek provenances under different stress concentrations served as the basis for a comprehensive assessment. The membership function method was applied to calculate a comprehensive evaluation D-value for each provenance, and the mean values were ranked to determine their relative tolerance.

The findings revealed that at stress concentrations of 0, 50, and 100 mmol/L, the Qinghai fenugreek achieved the highest comprehensive D-values (0.6413, 0.5792, and 0.6102, respectively), followed by the Anhui fenugreek (0.5634, 0.5788, and 0.5952). Under higher stress levels of 150 and 200 mmol/L, the Anhui fenugreek exhibited the highest D-values (both 0.5980), with the Qinghai provenance ranking second (0.5729 and 0.4684). Consequently, the overall saline-alkali tolerance, in descending order, was as follows: Qinghai fenugreek (0.5744) > Anhui fenugreek (0.5621) > Yunnan fenugreek (0.5260) > Gansu fenugreek (0.4992) (**Table 7**).

**Table 7:** Comprehensive evaluation of saline-alkali tolerance of four fenugreek materials under saline-alkali stress.

Provenance	Salt concentration	Comprehensive evaluation of D value	Mean D value	Sort
Qinghai fenugreek	0	0.6413	0.5744	1
	50	0.5792		
	100	0.6102		
	150	0.5729		
	200	0.4684		
Ganshu fenugreek	0	0.5172	0.4992	4
	50	0.4779		
	100	0.5637		
	150	0.5367		
	200	0.4006		
Yunnan fenugreek	0	0.5494	0.5260	3
	50	0.5212		
	100	0.5640		
	150	0.5701		
	200	0.4255		
Anhui fenugreek	0	0.5634	0.5621	2
	50	0.5788		
	100	0.5952		
	150	0.5980		
	200	0.4751		

### 3.5 Quality assessment and analysis of total RNA from transcriptome sequencing samples

Comprehensive assessments of growth, physiological revealed that the overall tolerance at the seedling stage followed the order: Qinghai provenance> Anhui provenance> Yunnan provenance> Gansu provenance. Consequently, the Qinghai provenance, which exhibited the greatest tolerance, was selected for transcriptome analysis. The control group was designated as QH CK, while the groups treated with 100 mmol/L and 200 mmol/L stress were designated as QH 100 and QH 200, respectively.

**Table 8** presents the quality evaluation of total RNA extracted from fenugreek samples. All nine samples met the criteria for library preparation, with RNA concentrations exceeding 250 ng/μl, RNA integrity number (RIN) values of at least 6.5, OD _260/280_ ratios between 2.1 and 2.2, OD _260/230_ ratios ranging from 1.06 to 2.1, and 28 S/18 S ratios above 1.5. These findings demonstrate that the total RNA from the nine fenugreek samples had high integrity and high purity.

**Table 8:** The detection results of total RNA quality.

Sample name	Concentration (ng/μl)	RIN value	28 S/18 S	OD _260/280_	OD _260/230_
QH CK-1	364	6.9	2.54	2.16	1.71
QH CK-2	277.7	7.7	1.73	2.16	1.31
QH CK-3	355.2	7.6	2.55	2.1	1.88
QH 100-1	477.9	7.7	2.43	2.15	2.02
QH 100-2	267.9	7.9	2.07	2.14	1.63
QH 100-3	365.9	7.9	2.15	2.17	1.06
QH 200-1	372.4	7.9	2.27	2.15	1.88
QH 200-2	477.4	7.5	1.64	2.2	1.13
QH 200-3	299.2	7.7	1.89	2.13	2.1

### 3.6 Transcriptome sequencing and assembly

**Figure 6:**
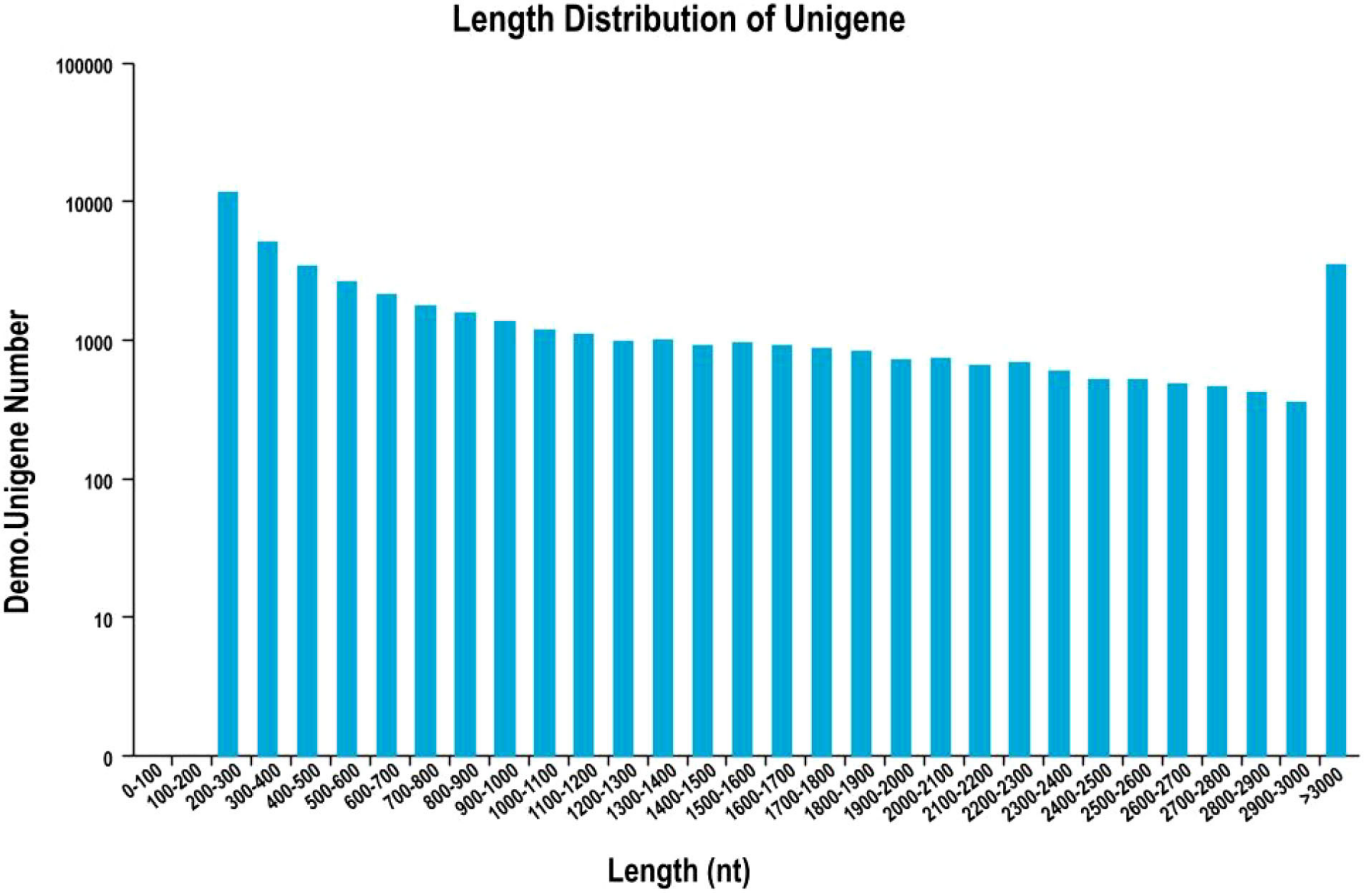
Unigene length distribution.

After quality control of the sequencing data, a total of 64.45 Gb of Clean Data was obtained from the nine samples. The percentage of bases with a Q30 quality score ranged from 94.17% to 95.46%, and the GC content for all samples was greater than 40.00% (**Table 9**). Following the acquisition of high-quality sequencing data, *de novo* assembly was conducted using Trinity software (**Figure 6**; **Table 10**). The assembly yielded 104,657 transcripts, with an N50 length of 1,992 bp and an average length of 1,305.57 bp. From these transcripts, 47,757 unigenes were obtained, exhibiting an N50 length of 2,017 bp and an average length of 1,125.14 bp. The length distribution of the unigenes was as follows:11,612 unigenes (24.31%) were 200–300 bp in length;8,467 unigenes (17.73%) were 300–500 bp in length;9,389 unigenes (19.66%) were 500-1,000 bp in length;9,432 unigenes (19.75%) were 1,000-2,000 bp in length;8,857 unigenes (18.55%) were longer than 2,000 bp.

**Table 9:** Overview of the high-throughput sequencing data.

Sample name	Read number	Clean data base number	GC content(%)	%≥Q30
QH CK-1	23936298	7166068428	42.27%	94.53%
QH CK-2	24845857	7427792906	42.15%	94.24%
QH CK-3	23287273	6966522815	41.42%	94.17%
QH 100-1	23219988	6927865541	42.30%	94.31%
QH 100-2	24645473	7371158347	41.62%	94.48%
QH 100-3	24722093	7384637824	42.22%	94.41%
QH 200-1	23774741	7090806218	42.38%	94.48%
QH 200-2	22495389	6734112074	42.51%	95.46%
QH 200-3	24686480	7377255589	42.22%	94.60%

**Table 10:** Length distribution of assembled transcripts and unigenes.

Length range	Transcript	Unigene (article)
200 ~ 300	15,061(14.39%)	11,612(24.31%)
300 ~ 500	14,985(14.32%)	8,467(17.73%)
500 ~ 1000	22,804(21.79%)	9,389(19.66%)
1000 ~ 2000	29,172(27.87%)	9,432(19.75%)
2000+	22,635(21.63%)	8,857(18.55%)
Total Number	104657	47757
Total Length	136951194	53733451
N50 Length	1992	2017
Mean Length	1308.57	1125.14

To further evaluate the reproducibility among biological replicates and the overall transcriptional differences among treatments, we performed principal component analysis (PCA) using variance-stabilized (VST) normalized read counts. Samples generally clustered according to treatment (QHCK, QH100, and QH200), indicating good within-group consistency and clear separation between control and saline-alkali stress conditions. One replicate in the QH100 group showed a modest deviation along PC2, suggesting some within-group variability; nevertheless, the overall clustering pattern remained treatment-dependent (**Figure 4**).

### 3.7 Functional annotation of unigenes

In this study, BLAST and HMMER searches were performed with E-value cutoffs of ≤1e^−5^ and ≤1e^−10^ (Eli, 2019), respectively. A total of 47,757 assembled unigenes were aligned against multiple public databases, including NR, Swiss-Prot, COG, KOG, eggNOG 4.5, and KEGG (**Table 11**). Consequently, 27,300 unigenes were successfully annotated, yielding an annotation rate of 57.16%. The detailed annotation results are presented below: COG: 6,370 unigenes (23.33%); GO: 22,183 unigenes (81.26%); KEGG: 16,697 unigenes (61.16%); KOG: 13,320 unigenes (48.79%); Pfam: 17,809 unigenes (65.23%); Swiss-Prot: 16,319 unigenes (59.78%); TrEMBL: 26,577 unigenes (97.35%); eggNOG: 21,403 unigenes (78.40%); NR: 26,487 unigenes (97.02%). Percentages indicate the proportion of annotated unigenes that were assigned to each specific database. 7,541 unigenes ranged from 300 to 1,000 bp, while 16,492 unigenes were ≥1,000 bp in length.

**Table 11:** Unigene annotation statistics of fenugreek.

Anno-Database	Number of annotated unigenes	Number of Unigenes annotated to this bin	
		300≤Length<1000	Length≥1000
COG_Annotation	6370	961	5114
GO_Annotation	22183	5562	14286
KEGG_Annotation	16697	3726	11553
KOG_Annotation	13320	2727	9349
Pfam_Annotation	17809	3636	13090
Swissprot_Annotation	16319	3427	11733
TrEMBL_Annotation	26577	7255	16394
eggNOG_Annotation	21403	5194	14127
nr_Annotation	26487	7188	16355
All_Annotated	27300	7541	16492

**Figure 7:**
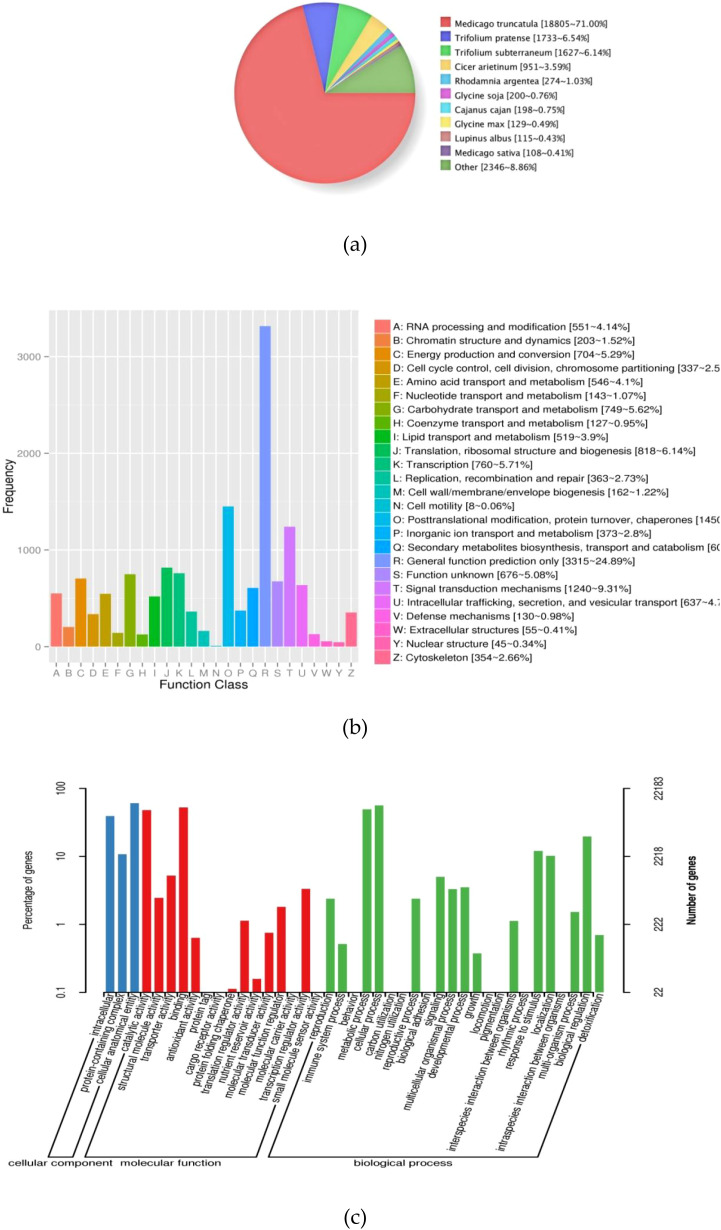
**(a)** NR-annotated species distribution map similar to the fenugreek transcriptome. **(b)** functional classification of KOG annotated homologous sequences. **(c)** GO annotation function classification.

The NR database is a non-redundant protein database within NCBI, which integrates protein sequences from GenBank, EMBL, DDBJ, and PDB (Pruitt et al., 2007). A relatively large number of unigenes (26,487) were annotated against the NR database. Based on the results of the smallest BLAST E-values, fenugreek showed the highest sequence similarity to *Medicago truncatula* Gaertn. (71.00%), followed by *Trifolium pratense* L. (6.54%) and *Trifolium subterraneum* L. (6.14%) (**Figure 7a**).

KOG classification is based on orthologous and evolutionary relationships of genes (Koonin et al., 2004). A total of 13,320 unigenes were categorized into 25 functional groups (**Figure 7b**). The functional categories representing more than 5.00% of the annotated unigenes were as follows: General Function Prediction Only (3,315 unigenes, 22.29%); Posttranslational Modification, Protein Turnover, and Chaperones (1,450 unigenes, 9.75%); Signal Transduction Mechanisms (1,240 unigenes, 8.35%); Translation, Ribosomal Structure and Biogenesis (818 unigenes, 5.50%); Transcription (760 unigenes, 5.11%); Carbohydrate Transport and Metabolism (749 unigenes, 5.04%). Conversely, the three categories containing the fewest annotated unigenes were: Extracellular Structures (55 unigenes, 0.37%); Nuclear Structure (45 unigenes, 0.30%); Cell Motility (8 unigenes, 0.05%).

The GO database is organized into three primary domains: Molecular Function, Cellular Component, and Biological Process (Ashburner et al., 2000). A total of 22,183 unigenes were assigned to 41 GO terms (**Figure 7c**), distributed across 23 biological processes, 15 molecular functions, and 3 cellular components. The top three GO terms within the molecular function domain were: Binding (11,695 unigenes, 52.72%); Catalytic Activity (10,677 unigenes, 48.13%); Transporter Activity (1,157 unigenes, 5.22%). All three annotated GO terms within the cellular component domain were: Cellular Anatomical Entity (13,486 unigenes, 60.79%); Intracellular (8,703 unigenes, 39.23%); Protein-containing Complex (2,392 unigenes, 10.78%). The leading GO terms within the biological process domain were: Cellular Process (12,518 unigenes, 56.43%); Metabolic Process (10,940 unigenes, 49.32%); Biological Regulation (4,362 unigenes, 19.66%).

### 3.8 Gene expression analysis

**Figure 8:**
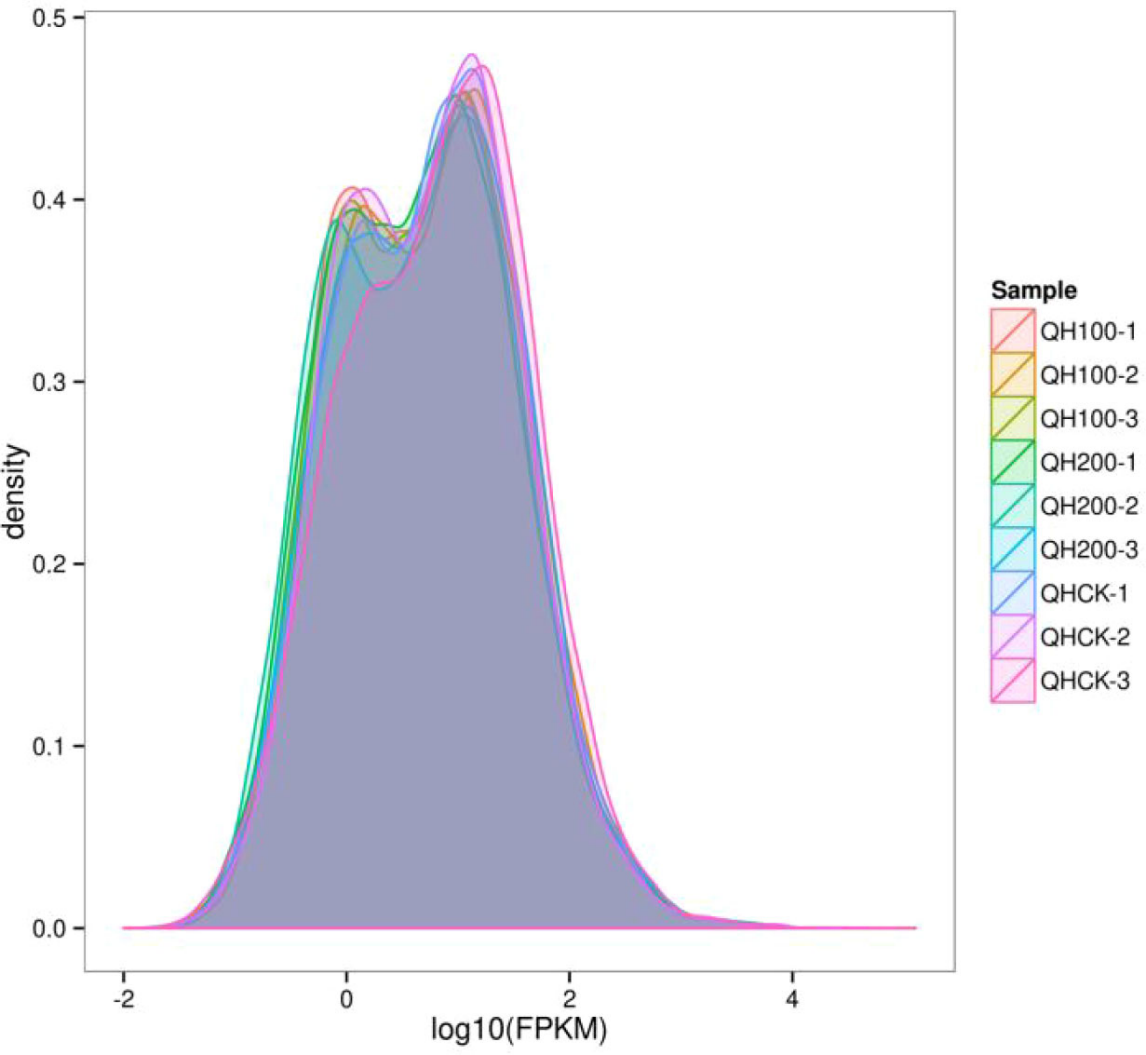
FPKM density distribution comparison diagram of fenugreek samples.

Transcriptome data demonstrated high sensitivity for detecting gene expression. **Figure 8** shows the overall distribution of gene expression levels in fenugreek. The FPKM values of the detected protein-coding genes spanned six orders of magnitude, from 10–^2^ to 10^4^ (CNCB-NGDC Members and Partners, 2025).

### 3.9 Analysis of differential gene expression under saline-alkali stress in fenugreek

#### 3.9.1 Screening of differentially expressed genes

**Figure 9:**
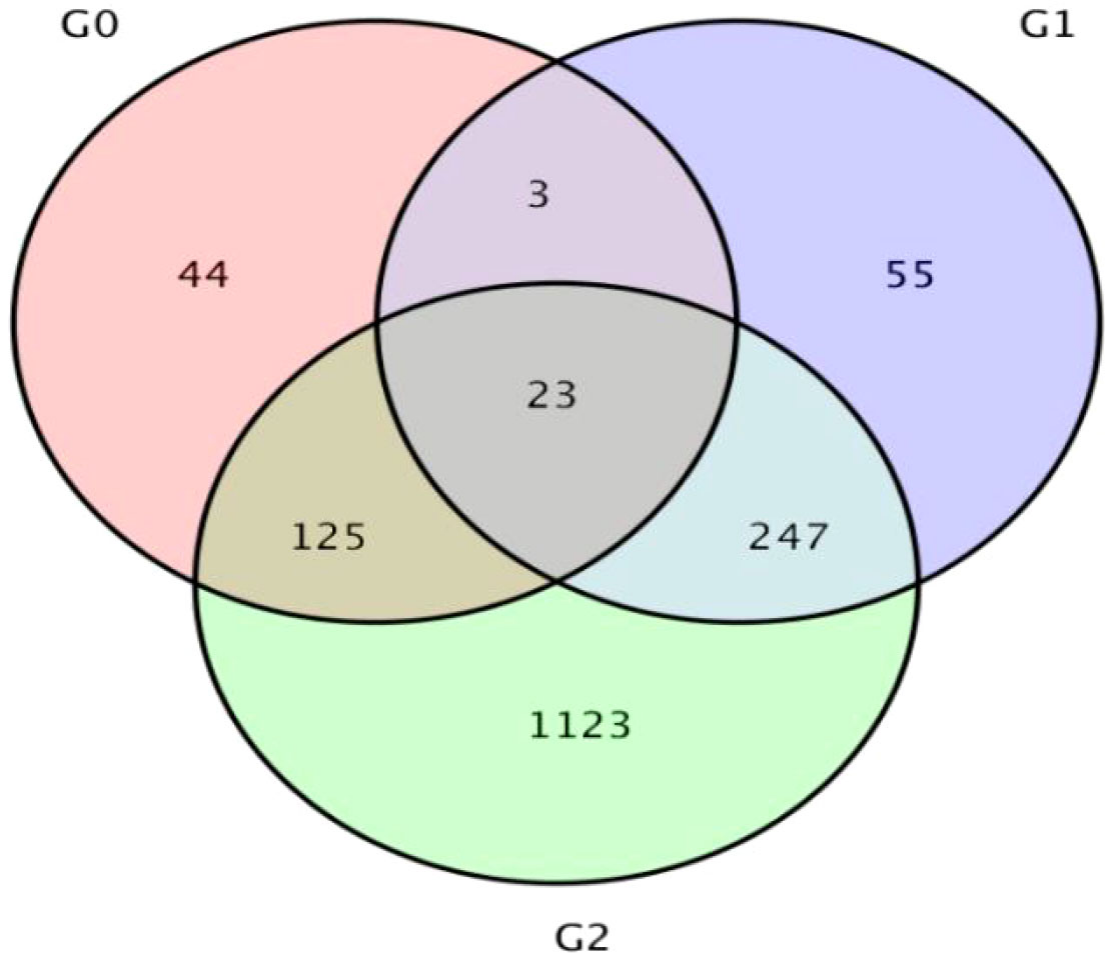
Analysis of differentially expressed genes between treatment groups. G0, G1, and G2 represent the comparison groups QH 100 vs QH 200, QH CK vs QH 100, and QH CK vs QH 200, respectively.

Genes exhibiting significant differences in expression levels between samples are defined as differentially expressed genes (DEGs). The analysis yielded three DEG sets: the QH 100 vs QH 200 comparison revealed 195 DEGs (138 upregulated and 57 downregulated); the QH CK vs QH 100 comparison identified 328 DEGs (139 upregulated and 189 downregulated); and the QH CK vs QH 200 comparison detected 1,518 DEGs (799 upregulated and 719 downregulated). These results indicate that the number of DEGs increased with the intensity of saline-alkali stress. Across the three comparisons, a total of 1,620 unique DEGs were identified, with some overlap among the DEG sets. Notably, 23 genes were differentially expressed in all three comparison groups (**Table 12**; **Figure 9**).

**Table 12:** The number of differentially expressed genes.

DEG_Set	All_DEG	Up-regulated	Down-regulated
QH 100 vs QH 200	195	138	57
QH CK vs QH 100	328	139	189
QH CK vs QH 200	1518	799	719

#### 3.9.2 Functional enrichment analysis of differentially expressed genes

A total of 188, 315, and 1,466 DEGs were annotated for the QH 100 vs QH 200, QH CK vs QH 100, and QH CK vs QH 200 comparisons, respectively. Across these three treatment comparisons, a substantial number of DEGs were assigned to major functional databases, including GO, KEGG, NR, and eggNOG. This result suggests that saline-alkali stress induced the expression of a wide range of functional genes in fenugreek (**Table 13**).

**Table 13:** Statistical annotation of differentially expressed genes between different treatments.

DEG Set	Total	COG	GO	KEGG	KOG	NR	Pfam	Swiss-Prot	eggNOG
QH 100 vs QH 200	188	68	155	131	80	188	155	153	162
QH CK vs QH 100	315	101	262	209	149	315	248	235	263
QH CK vs QH 200	1466	536	1274	990	701	1464	1182	1141	1257

##### 3.9.2.1 GO classification and enrichment analysis of DEGs

The GO database is structured into three primary ontologies: Biological Process, Molecular Function, and Cellular Component. Based on the gene annotation results, the differentially expressed genes were categorized and statistically analyzed at the secondary level of the GO hierarchy.

**Figure 10:**
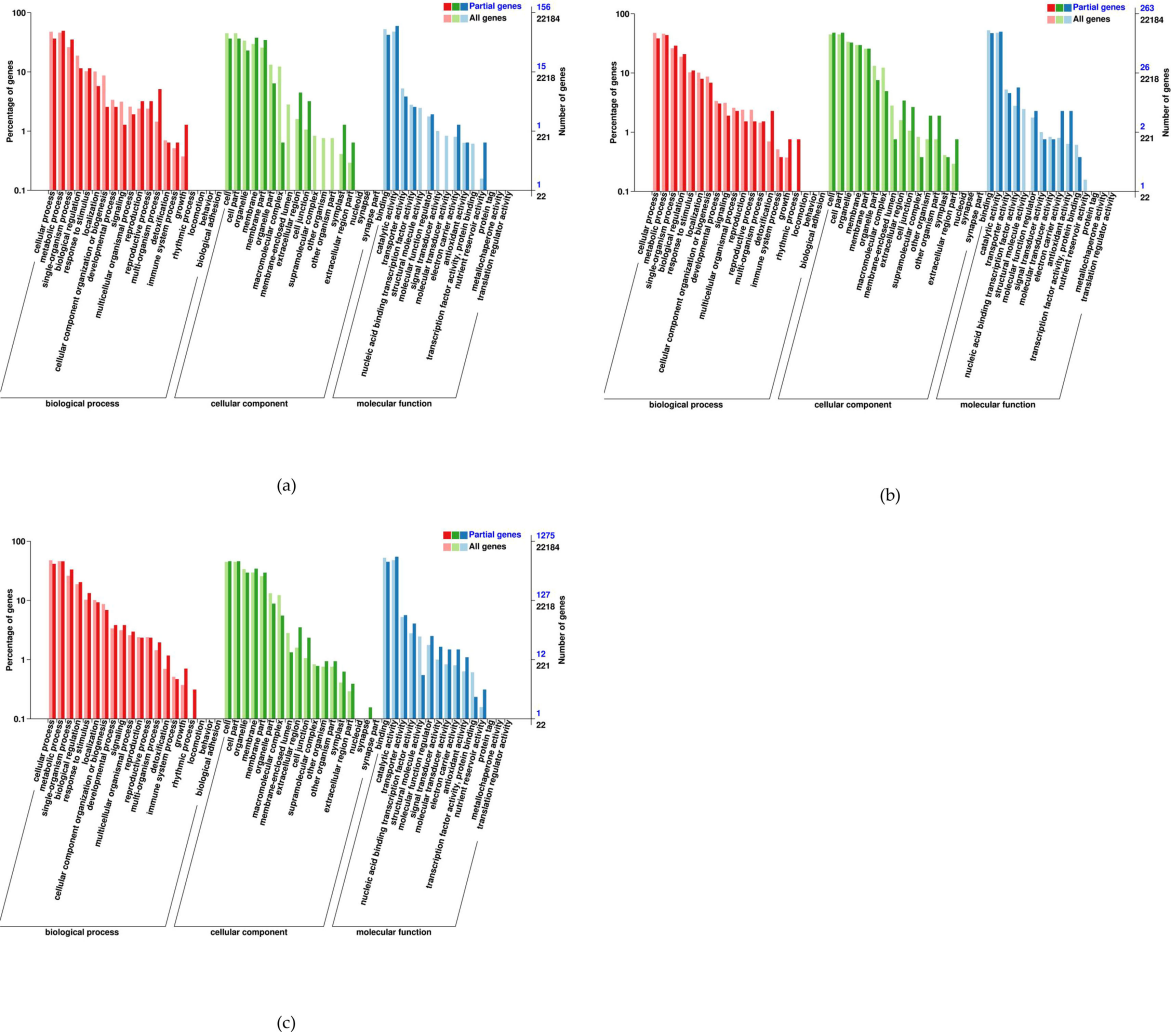
Gene Ontology functional analysis of DEGs: **(a)** QH 100 vs QH 200; **(b)** QH CK vs QH 100; **(c)** QH CK vs QH 200.

GO annotation was conducted for the DEGs identified in the comparisons QH 100 vs QH 200, QH CK vs QH 100, and CK vs QH 200, which included 188, 315, and 1,466 genes respectively. Of these, 155, 262, and 1,274 genes were successfully annotated (**Figure 10**). Within the Biological Process category, the majority of DEGs were linked to Metabolic Process, Cellular Process, Response to Stimulus, Biological Regulation, Localization, and Multi-organism Process, suggesting these processes play key roles in the saline-alkali tolerance of fenugreek. Regarding Molecular Function, a large portion of DEGs were associated with Catalytic Activity, Binding, Transporter Activity, and Transcription Regulator Activity. In the Cellular Component category, DEGs were predominantly enriched in Cellular Anatomical Entity, Intracellular, and Protein-containing Complex, indicating that saline-alkali stress induces substantial alterations in plant cells and protein complexes.

##### 3.9.2.2 KEGG annotation and pathway enrichment analysis of DEGs

**Figure 11:**
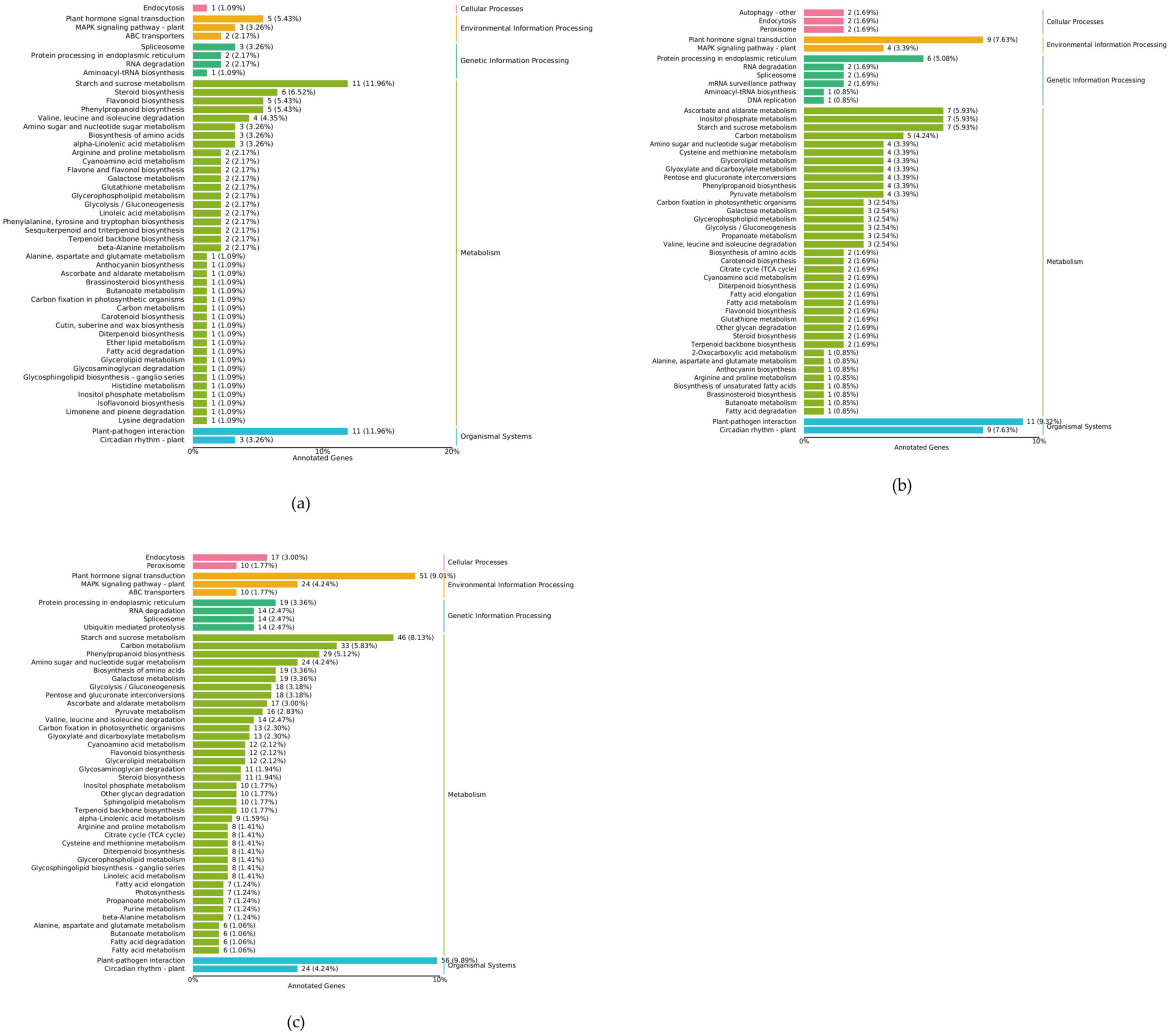
Differentially expressed genes KEGG annotation and pathway enrichment scatter plot: **(a)** QH 100 vs QH 200; **(b)** QH CK vs QH 100; **(c)** QH CK vs QH 200.

The KEGG database is a knowledge base for the systematic analysis of gene functions and genomic information. It provides comprehensive annotations of enzymes catalyzing each reaction step across all known metabolic pathways, serving as a powerful tool for *in vivo* metabolic analysis and metabolic network research (Kanehisa et al., 2004). **Figure 11a** (displaying only the top 20 pathways) shows the KEGG pathway enrichment results for the DEGs from the QH 100 vs QH 200 comparison in fenugreek. In total, 131 genes were annotated and mapped to 71 pathways. The most significantly enriched pathways included Flavonoid biosynthesis, Plant hormone signal transduction, Plant-pathogen interaction, Starch and sucrose metabolism, Steroid biosynthesis, and Phenylpropanoid biosynthesis. This suggests that these pathways were actively involved in the response to saline-alkali stress.

**Figure 11b** presents the KEGG pathway enrichment analysis for DEGs from the QH CK vs QH 100 comparison in fenugreek. A total of 209 genes were annotated, covering 83 pathways. The DEGs were significantly enriched in pathways such as Circadian Rhythm - plant, Starch and Sucrose Metabolism, Plant-pathogen Interaction, Plant Hormone Signal Transduction, Ascorbate and Aldarate Metabolism, Inositol Phosphate Metabolism, and Protein Processing in Endoplasmic Reticulum. This suggests that these pathways play significant roles in the saline-alkali stress response of fenugreek.

**Figure 11c** shows the KEGG pathway enrichment analysis for DEGs from the QH CK vs QH 200 comparison in fenugreek. Out of 1,466 DEGs, 990 were successfully mapped to 122 KEGG pathways. The pathways with the highest number of mapped DEGs were plant-pathogen interaction (56 genes), plant hormone signal transduction (51 genes), and starch and sucrose metabolism (46 genes). Additionally, other pathways such as phenylpropanoid biosynthesis, carbon metabolism, MAPK signaling pathway - plant, circadian rhythm - plant, and amino sugar and nucleotide sugar metabolism were also notably enriched. These findings suggest that the response of fenugreek to saline-alkali stress primarily involves genes participating in these metabolic and signaling pathways.

##### 3.9.2.3 COG annotation of DEGs

**Figure 12:**
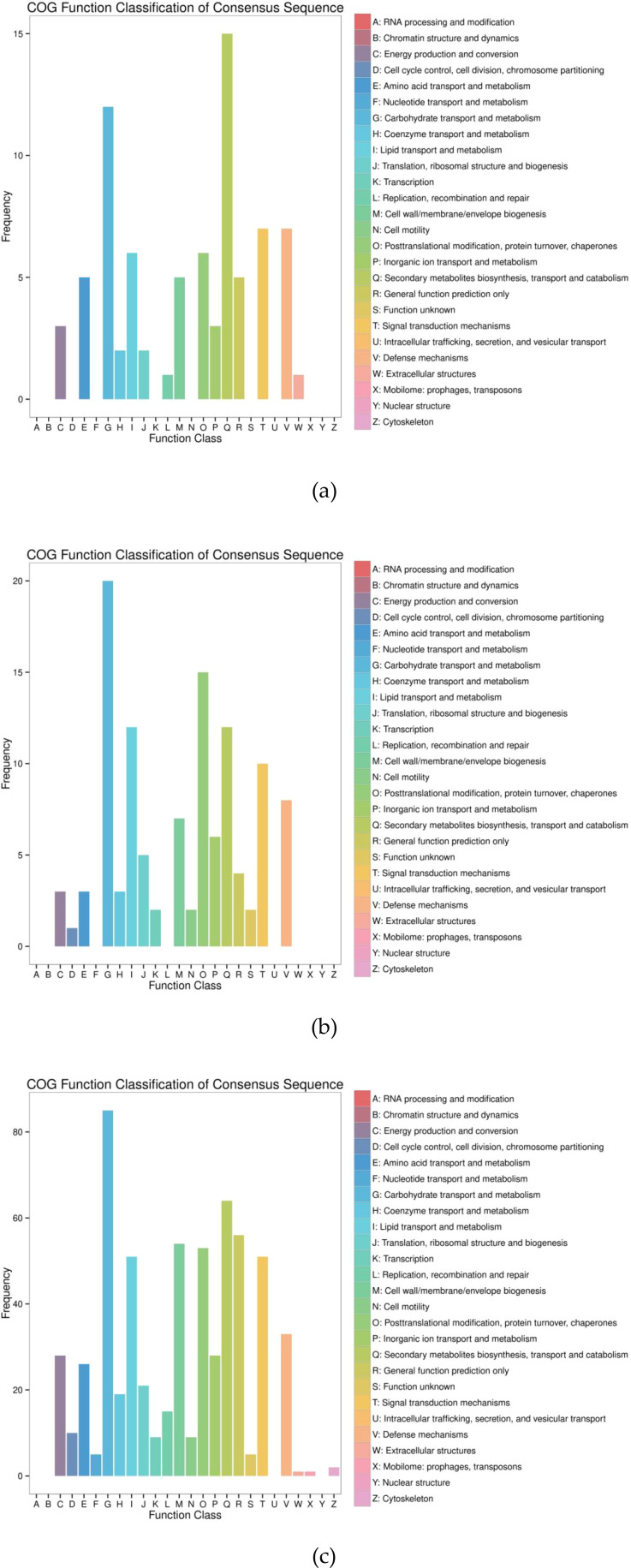
COG annotation clustering figure of DEG (continued): **(a)** QH 100 vs QH 200; **(b)** QH CK vs QH 100; **(c)** QH CK vs QH 200.

COG (Clusters of Orthologous Groups) is a database for the homologous classification of gene products, which are functionally divided into 26 categories. It allows for the classification of gene products based on orthology (Tatusov et al., 2000). Among the 188 DEGs in the QH 100 vs QH 200 comparison, 68 genes were annotated in the COG database and classified into 15 functional categories (**Figure 12a**). The largest category was Secondary metabolites biosynthesis, transport and catabolism, accounting for 22.06% of the COG-annotated genes. This was followed by Carbohydrate transport and metabolism (17.65%), Defense mechanisms (10.29%), and Signal transduction mechanisms (10.29%). In contrast, the categories Replication, recombination and repair and Extracellular structures contained the fewest genes, with only one gene annotated in each category.

In the QH CK vs QH I00 comparison, 101 DEGs were annotated in the COG database and classified into 17 functional categories (**Figure 12b**). The most prominent categories were carbohydrate transport and metabolism and posttranslational modification, protein turnover, chaperones, comprising 19.80% and 14.85% of the total annotated DEGs, respectively. The next most represented categories were lipid transport and metabolism and secondary metabolites biosynthesis, transport, and catabolism, each accounting for 11.88% of the COG-annotated genes. Furthermore, signal transduction mechanisms (9.90%), defense mechanisms (7.92%), cell wall/membrane/envelope biogenesis (6.93%), and inorganic ion transport and metabolism (5.94%) were also significantly represented. In contrast, the category cell cycle control, cell division, chromosome partitioning contained the fewest genes, with only a single gene annotated.

In the QH CK vs QH 200 comparison, 536 DEGs were annotated in the COG database and classified into 22 functional categories (**Figure 12c**). The most abundant category was carbohydrate transport and metabolism, comprising 85 DEGs. Several other categories were also well-represented, each with more than 50 annotated DEGs: General function prediction only; Secondary metabolites biosynthesis, transport, and catabolism; Cell wall/membrane/envelope biogenesis; Posttranslational modification, protein turnover, chaperones; Signal transduction mechanisms; Lipid transport and metabolism. Conversely, the categories extracellular structures and mobilome: prophages, transposons contained the fewest genes, with only a single gene annotated in each.

##### 3.9.2.4 Analysis of differentially expressed transcription factors

**Figure 13:**
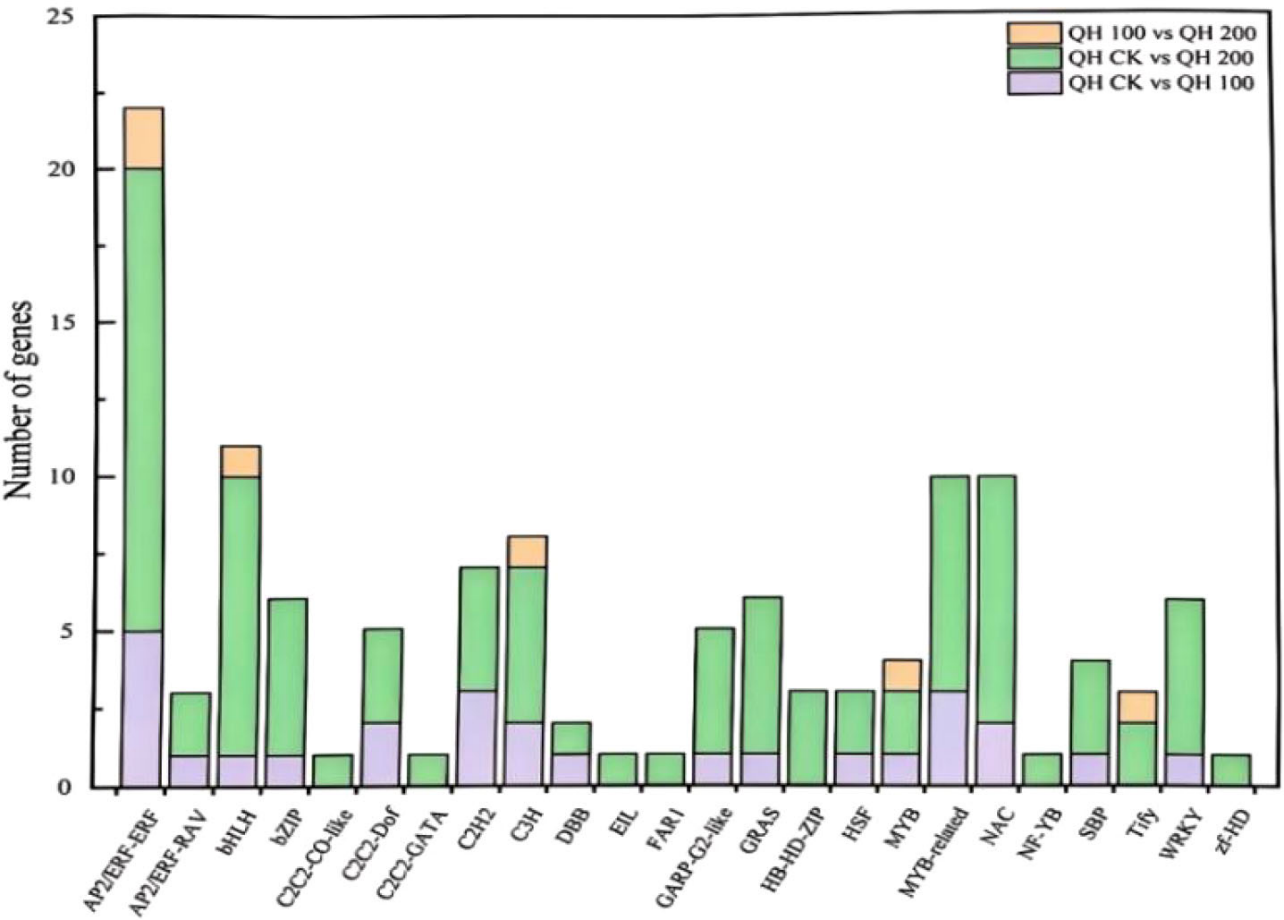
Analysis of differentially expressed transcription factors in fenugreek seedlings undermixed saline-alkali stress.

Transcription factors (TFs) play a crucial role in regulating gene expression at the molecular level in plants subjected to saline-alkali stress. They function by binding to cis-acting elements in the promoters of target genes (Fang et al., 2021). Utilizing the PlantTFDB database via the BMK cloud platform, we identified 27, 91, and 6 transcription factors responsive to abiotic stress in the QH CK vs QH 100, QH CK vs QH 200, and QH 100 vs QH 200 comparisons, respectively (**Figure 13**). These TFs were classified into 24 distinct families. The AP2/ERF-ERF family was the most prevalent, containing 22 members, with 8 upregulated and 14 downregulated. Additionally, TFs from the bHLH, MYB-related, NAC, and WRKY families were also notably represented, with 11, 10, and 10 members, respectively. The majority of these TFs were downregulated under saline-alkali stress conditions.

### 3.10 qRT-PCR validation

**Figure 14:**
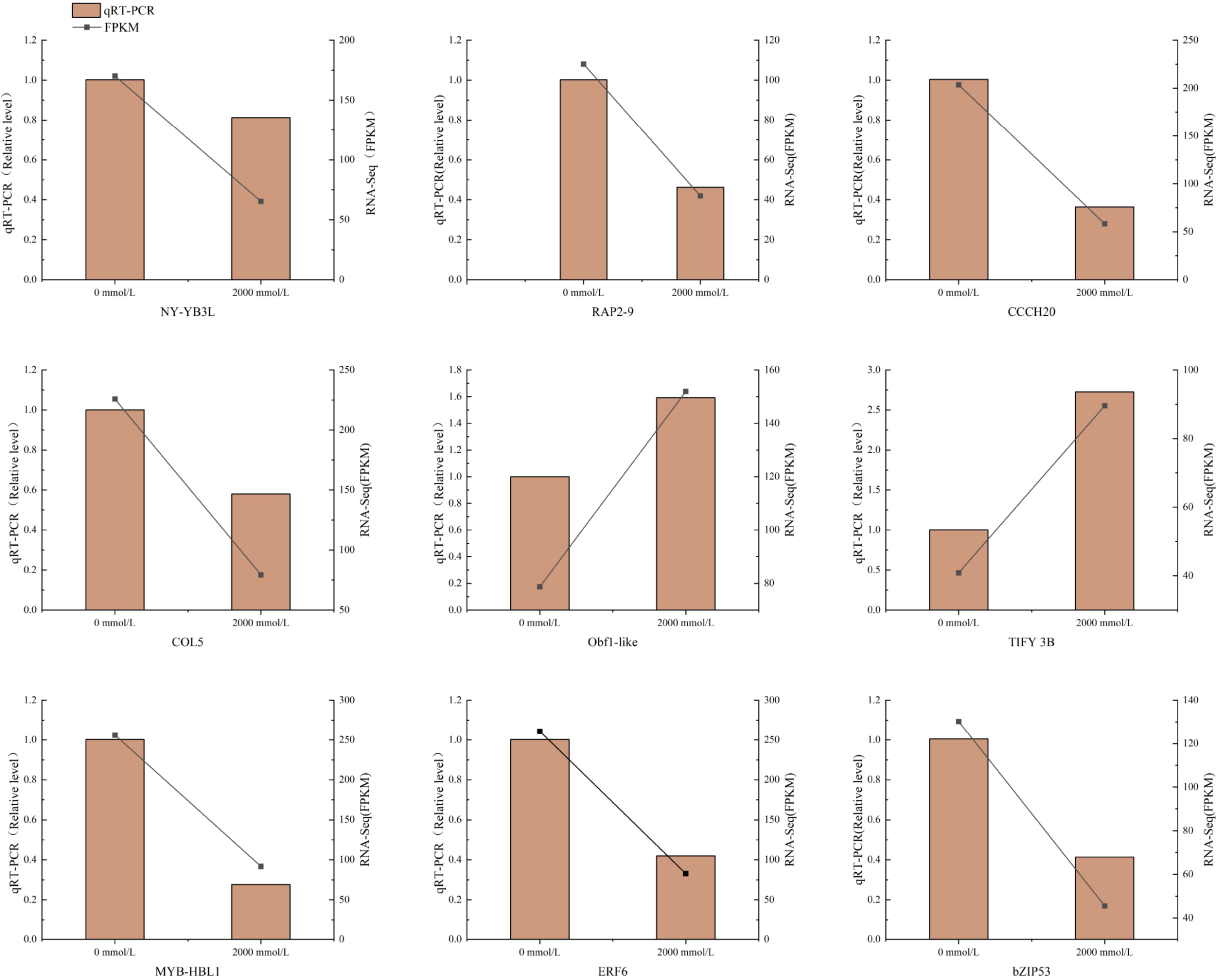
Validation of nine DEGs by qRT-PCR.

Transcription factor families such as bZIP, bHLH, MYB, WRKY, AP2, NAC, DREB, and C2H2 have been shown to play roles in plant responses to abiotic stresses (Luo et al., 2016). To validate the reliability of the RNA-Seq data, nine candidate genes related to saline-alkali stress were selected for qRT-PCR analysis. These candidate genes include those encoding zinc finger proteins, OBF proteins, nuclear transcription factors, ethylene-responsive transcription factors, MYB transcription factors, and bZIP transcription factors. Specifically, four are involved in signal transduction: *RAP2-9*, *Obf1-like*, *TIFY 3B*, and *ERF6*; four function in transcriptional regulation: *NF-YB3L*, *COL5*, *MYB-HBL1*, and *bZIP53*; and one is associated with RNA metabolism and post-translational modification: *CCCH20* (**Table 14**). The expression trends revealed by qRT-PCR were highly consistent with the RNA-Seq data, demonstrating the reliability of the transcriptome sequencing results obtained in this study (**Figure 14**).

**Table 14:** Annotation of candidate gene.

Functional category	Gene ID	Annotation information	Regulated		qRT-PCR
Signal transduction	*RAP2-9*	ethylene-responsive transcription factor RAP2–9 [*Medicago truncatula*]	down	down	
	*Obf1-like*	ocs element-binding factor 1-like protein [*Trifolium pratense*]	up	up	
	*TIFY 3B*	protein TIFY 3B [*Medicago truncatula*]	up	up	
	*ERF6*	ethylene-responsive transcription factor 6 [*Medicago truncatula*]	down	down	
Transcriptional regulation	*NF-YB3L*	nuclear transcription factor Y subunit B-3-like protein [*Trifolium pratense*]	down	down	
	*COL5*	zinc finger protein CONSTANS-LIKE 5 isoform X2 [*Medicago truncatula*]	down	down	
	*MYB-HBL1*	putative transcription factor MYB-HB-like family [*Medicago truncatula*]	down	down	
	*bZIP53*	bZIP transcription factor 53 [Medicago truncatula]	down	down	
RNA metabolism and post-translational regulation	*CCCH20*	zinc finger CCCH domain-containing protein 20 [*Medicago truncatula*]	down	down	

## 4 Discussion

Saline–alkali stress is a major abiotic constraint that impedes plant growth and development. Importantly, saline–alkali stress is a composite condition integrating both high-pH (alkaline) effects and salinity-driven osmotic and ionic stress. In our bicarbonate-dominated system (Na_2_CO_3_/Na_2_SO_4_/NaCl/NaHCO_3_ = 1:3:3:9), carbonate/bicarbonate elevates solution pH (**Table 2**), which may reduce nutrient availability and uptake and disturb cellular pH homeostasis, thereby triggering alkaline-related responses. Meanwhile, increasing total salt concentration increases ionic strength and Na^+^ load, leading to osmotic stress and ion imbalance. Accordingly, the accumulation of osmolytes (e.g., proline, soluble sugars, and soluble proteins) and the activation of antioxidant enzymes observed in this study can be interpreted primarily as adaptive responses to osmotic/ionic stress, while high pH likely imposes additional constraints on nutrient acquisition and cellular homeostasis.

Based on integrated growth, physiological, and photosynthetic traits, the four provenances exhibited a clear tolerance ranking under saline–alkali stress (Qinghai > Anhui > Yunnan > Gansu). The most tolerant Qinghai provenance was therefore selected for transcriptome profiling under 0, 100, and 200 mmol/L treatments.

Overall, the *de novo* transcriptome assembly generated 47,757 unigenes, of which 27,300 were functionally annotated across public databases (see Results, **Table 11**). This resource enabled downstream DEG identification and pathway interpretation under bicarbonate-dominated saline–alkali stress.

Transcriptome profiling revealed a stress-intensity–dependent transcriptional reprogramming, with increasing stress concentration, the number and diversity of DEGs increased significantly, and the number of upregulated genes was higher than that of downregulated genes. This trend is consistent with previous studies in *Populus davidiana* Dode × *Populus bolleana* Lauche (Lei et al., 2022), *Asparagus officinalis* L (Wen et al., 2024), and *Glycine soja* Siebold & Zucc (Lee et al., 2025). These findings suggest that, with increasing alkali stress, fenugreek seedlings employ more complex physiological and biochemical processes to cope with the adverse effects of saline-alkali conditions.

To elucidate the biological functions and signaling pathways of the DEGs, GO and KEGG enrichment analyses were performed. The GO enrichment results showed that, in terms of biological processes, DEGs were significantly enriched in cellular processes, metabolic processes, and biological regulation. For molecular functions, DEGs were predominantly enriched in binding, catalytic activity, and transporter activity. KEGG pathway analysis revealed that DEGs were enriched in pathways related to plant hormone signal transduction, starch and sucrose metabolism, and carbon metabolism. These findings are consistent with the transcriptome sequencing results of *Medicago sativa* L. reported by Li et al. (Li et al., 2019), suggesting that these pathways may be critical factors influencing saline-alkali tolerance in fenugreek.

TFs play crucial regulatory roles in gene expression under saline-alkali stress and can enhance plant tolerance by modulating the transcriptional levels of downstream target genes (Wan et al., 2015). Studies in Arabidopsis thaliana have identified over 1,500 TFs, including members of the bHLH, WRKY, MYB, NAC, and AP2/ERF families (Riechmann et al., 2000). Additionally, previous research has confirmed that transcription factor families such as bZIP, NAC, MYB, AP2, bHLH, and WRKY are closely associated with plant saline-alkali tolerance (Wen et al., 2024).

In this study, transcriptome sequencing of fenugreek seedlings identified 124 TFs responsive to abiotic stress, which belonged to 24 distinct TF families (**Figure 8**). Among these, members of the AP2/ERF-ERF, bHLH, MYB-related, and NAC families were the primary contributors to the saline-alkali stress response. These findings are consistent with the results of Ge et al. (2011) in *Medicago sativa* L.

Similarly, Ge et al. (2011) reported that members of the AP2-EREBP, bHLH, MYB/MYB-related, and C2C2-CO-like TF families accounted for a high proportion of the abiotic stress response in wild soybean. Zhu et al. (2021) demonstrated that the *WRKY75* gene enhances salt tolerance in transgenic peanut by increasing antioxidant enzyme activity and photosynthetic efficiency. In rice, overexpression of *ONAC022* improved drought and salt tolerance by regulating the ABA-mediated pathway (Hong et al., 2016). Zhou et al. (2020) found that heterologous overexpression of *CgbHLH001* in tobacco enhanced stress resistance by modulating the expression of stress-related genes.

The candidate genes identified in this study were annotated as encoding zinc finger proteins, OBF binding proteins, nuclear transcription factors, ethylene-responsive transcription factors, and MYB transcription factors. CCCH-type zinc finger proteins are widely present in eukaryotes and play important roles in plant growth, development, and responses to environmental stresses (Liu et al., 2024). Kong et al. (2006) reported that *OsDOS*, which encodes a CCCH-type zinc finger protein in rice, functions to delay leaf senescence. The zinc finger protein CONSTANS-LIKE (CO-like) belongs to the BBX subfamily of zinc finger proteins, characterized by the conserved B-box domain, and plays a crucial role in regulating plant growth, development, and responses to abiotic stress (Li et al., 2024). OBF proteins, members of the bZIP transcription factor family, are involved in plant responses to environmental stresses. Blume et al. (2000) found that OBF proteins regulate the expression of the *PR1* and *GST6* genes, thereby contributing to pathogen defense in parsley. Zhu et al. (2013) demonstrated that *GsTIFY* in soybean is upregulated under drought and salt stress, playing an important role in gene expression during growth, development, and defense. The expression patterns of the candidate genes detected by qRT-PCR were consistent with the RNA-Seq results.

**Figure 15:**
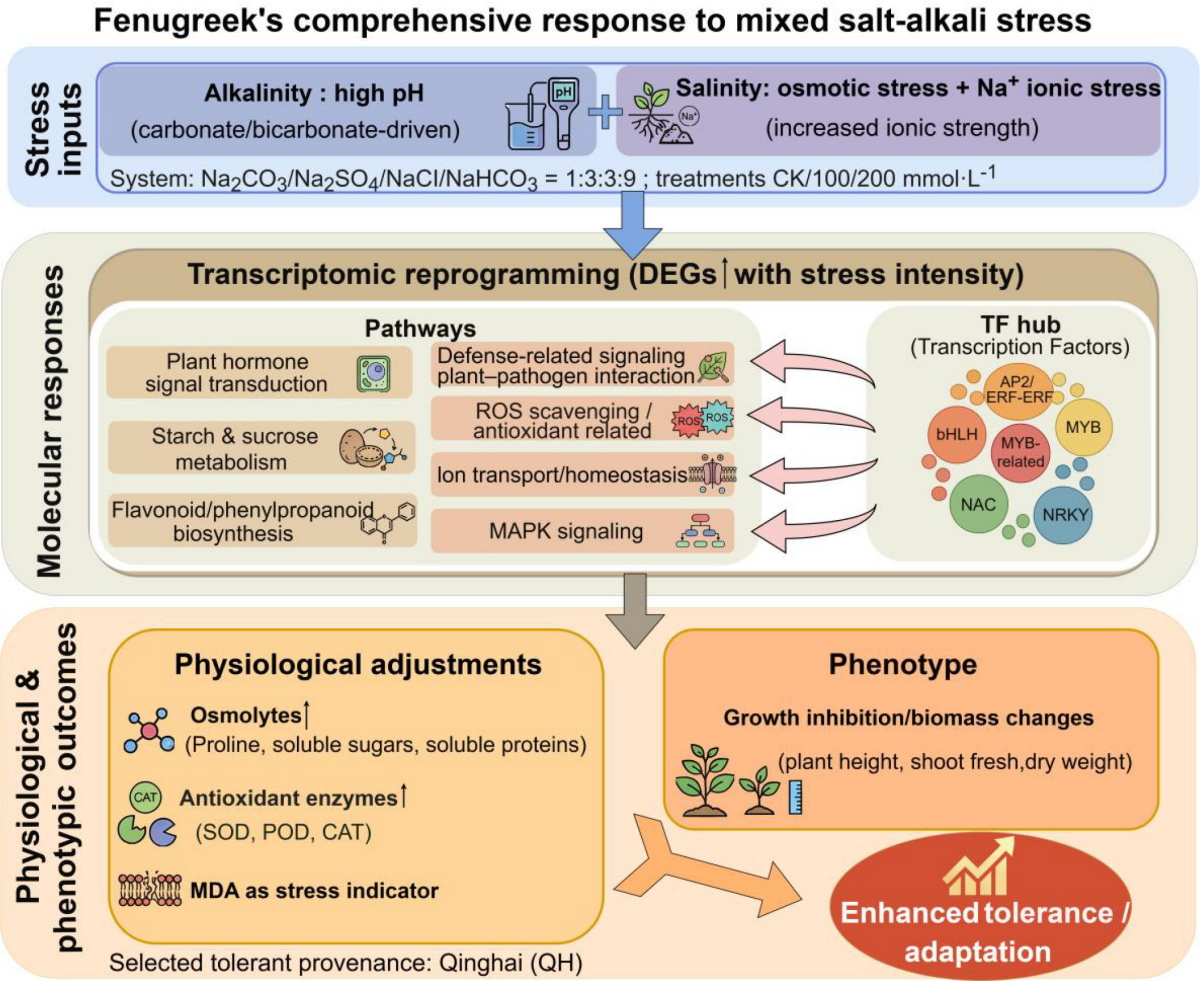
Proposed model of fenugreek responses to mixed saline–alkali stress.

To integrate the morpho-physiological measurements with transcriptomic responses, we propose a conceptual model summarizing fenugreek adaptation to mixed saline–alkali stress (**Figure 15**). The mixed saline–alkali treatment (Na_2_CO_3_:Na_2_SO_4_:NaCl: NaHCO_3_ = 1:3:3:9) imposes composite constraints comprising high-pH (alkaline) effects driven by carbonate/bicarbonate and salinity-driven osmotic/ionic effects associated with Na^+^ and accompanying anions. Transcriptomic changes (e.g., hormone signaling, carbohydrate metabolism, flavonoid biosynthesis, ROS scavenging, ion homeostasis and key transcription factor families) and morpho-physiological adjustments (growth/germination traits, osmolytes and antioxidant defense) together contribute to the integrated stress response and tolerance.

## 5 Conclusions

Following the screening of four fenugreek provenances, which identified the Qinghai accession as the most tolerant, this provenance was subjected to transcriptome analysis under a gradient of saline-alkali stress (0, 100, and 200 mmol/L). Transcriptome sequencing of fenugreek produced 47,757 unigenes, with 27,300 successfully annotated in public databases. The numbers of DEGs in the QH 100 vs QH 200, QH CK vs QH 100, and QH CK vs QH 200 comparisons were 195 (138 upregulated, 57 downregulated), 328 (139 upregulated, 189 downregulated), and 1,518 (799 upregulated, 719 downregulated), respectively. GO annotation indicated that the DEGs were predominantly enriched in terms related to metabolic process, cellular process, response to stimulus, and catalytic activity. KEGG pathway analysis revealed that the DEGs were significantly enriched in pathways including flavonoid biosynthesis, plant hormone signal transduction, starch and sucrose metabolism, and plant-pathogen interaction. Furthermore, 124 TFs associated with abiotic stress response were identified, spanning 24 TF families. Notably, members of the AP2/ERF-ERF, bHLH, MYB-related, and NAC families represented a significant proportion of these TFs.

## Data Availability

The raw sequence data reported in this paper have been deposited in the Genome Sequence Archive (Genomics, Proteomics & Bioinformatics 2021) in National Genomics Data Center (Nucleic Acids Res 2022), China National Center for Bioinformation/Beijing Institute of Genomics, Chinese Academy of Sciences (GSA: CRA030543) that are publicly accessible at https://ngdc.cncb.ac.cn/gsa.

## Glossary

DEGs: Differentially Expressed Genes
RNA-Seq: Transcriptome Sequencing
FDR: False Discovery Rate
RIN: RNA Integrity Number
TFs: Transcription Factors
GO: Gene Ontology
COG: Clusters of Orthologous Genes
KEGG: Kyoto Encyclopedia of Genes and Genomes
KOG: EuKaryotic Orthologous Groups
Pfam: Protein Families
Swissprot: Swiss-Prot Protein Knowledgebase
TrEMBL: Translated European Molecular Biology Laboratory Nucleotide Sequence Database
eggNOG: evolutionary genealogy of genes: Non-supervised Orthologous Groups
NR: Non-Redundant Protein Database
