# Supplementary material for: Transcriptome analysis of fenugreek under mixed saline-alkali stress
Source: Front Plant Sci. 2026 Mar 30;17:1753943. doi: 10.3389/fpls.2026.1753943 (PMC13070767; doi:10.3389/fpls.2026.1753943)
Supplement: Supplementary file 3 [file Supplementaryfile3.docx]

Supplementary Material

# Supplementary Figures and Tables

## Supplementary Figures


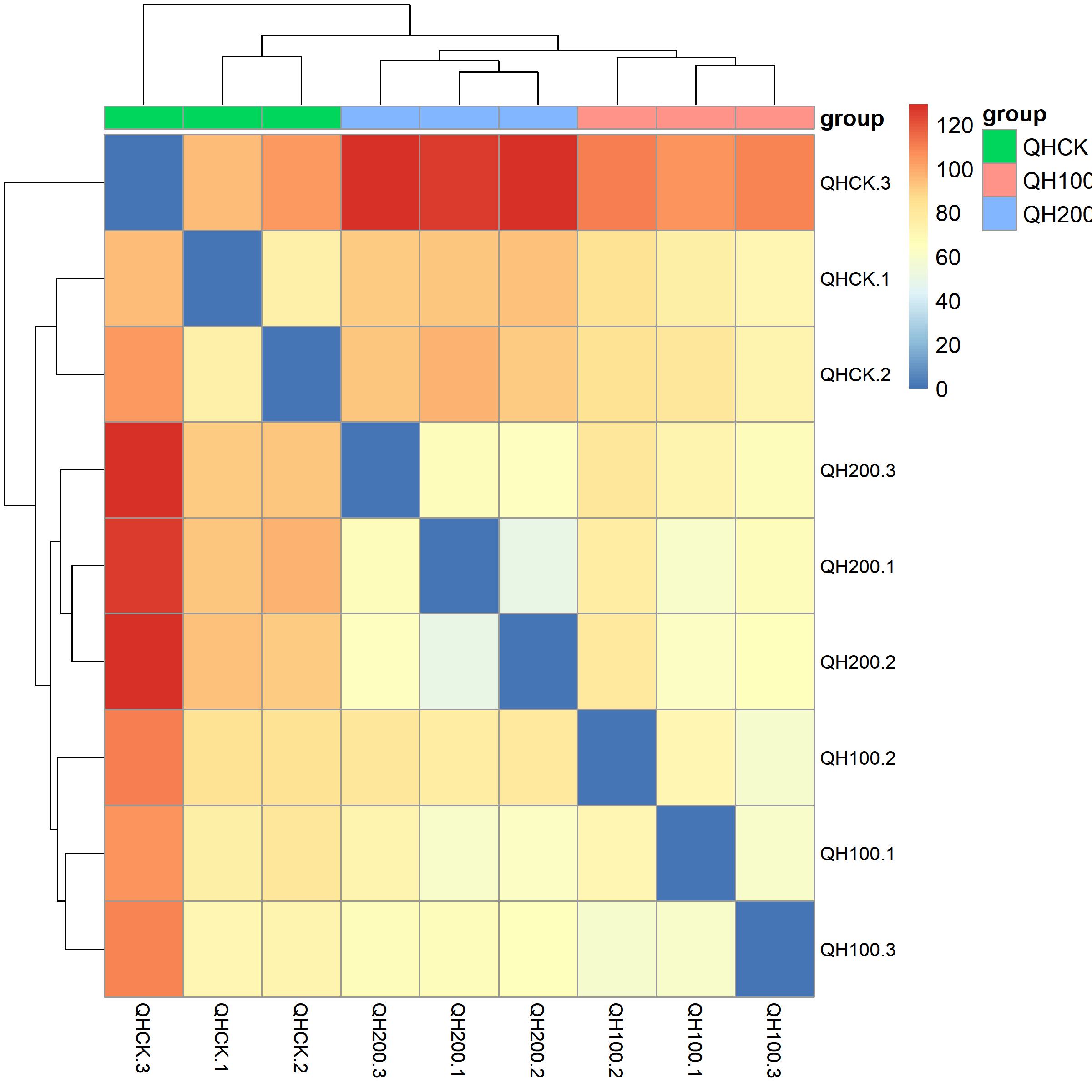


**Supplementary Figure 1.** Sample distance heatmap（VST）


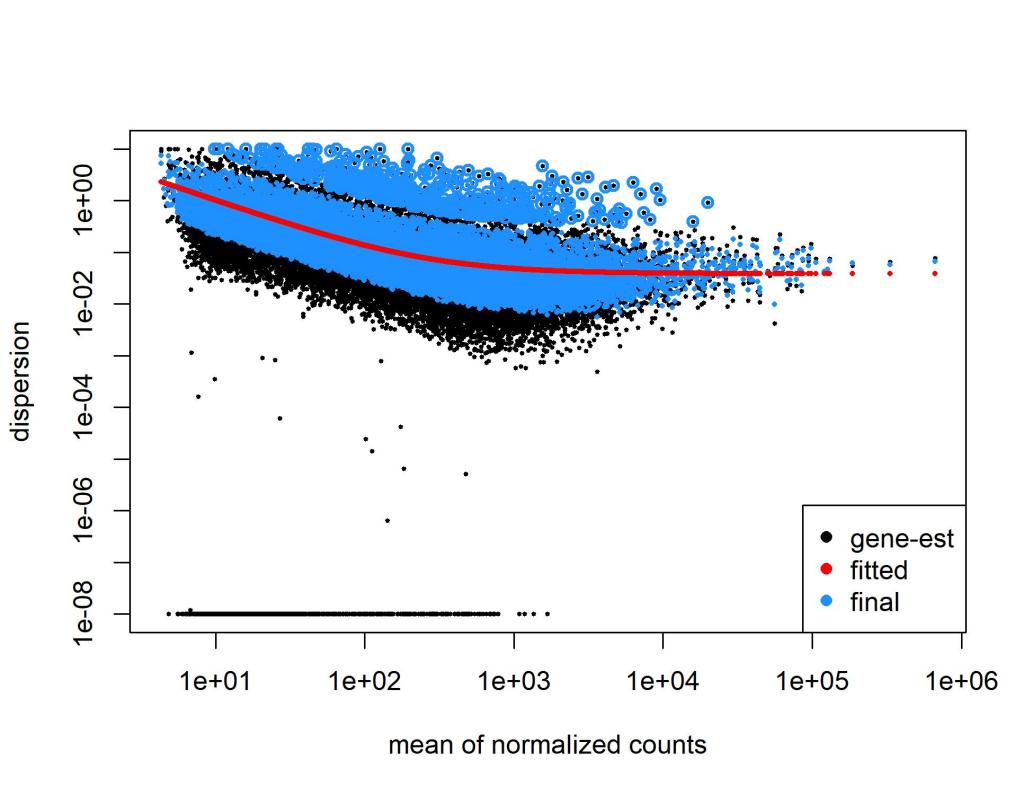


**Supplementary Figure 2.** Dispersion estimates plot（DESeq2）
